# Association between systemic immune-inflammation index and all-cause mortality in ischemic stroke patients

**DOI:** 10.3389/fneur.2026.1750717

**Published:** 2026-04-08

**Authors:** Junmou Li, Zhenwei Wang, Xuerong Sun, Lin Li, Zhuping Sun

**Affiliations:** 1 Department of Rehabilitation Medicine, Dandong Central Hospital, China Medical University, Dandong, Liaoning, China; 2 Department of Cardiology, The First Affiliated Hospital of Zhengzhou University, Zhengzhou, China

**Keywords:** all-cause mortality, ischemic stroke, prognostic biomarker, risk stratification, systemic immune-inflammation index

## Abstract

**Objectives:**

This study is designed to assess the association between the systemic immune-inflammation index (SII) and all-cause mortality among patients with ischemic stroke (IS).

**Methods:**

A single-center retrospective cohort study was conducted, including 1,156 IS patients discharged from Dandong Central Hospital from January to December 2024. The formula for SII is as follows: SII = (neutrophil count × platelet count)/lymphocyte count. Multivariate Cox regression model, subgroup analysis, sensitivity analysis, receiver operating characteristic (ROC) curve, and Kaplan–Meier survival analysis were used to evaluate the association between SII and all-cause mortality.

**Results:**

During the median follow-up period of 14.23 months, a total of 97 (8.4%) patients with all-cause mortality were identified. Multivariate Cox regression analysis showed that after adjusting for a variety of confounding factors, for every one-standard-deviation increase in SII, the risk of all-cause mortality increased by 17.2% [hazard ratio (HR) = 1.172, 95% confidence interval (CI): 1.019–1.348, *p* = 0.026]. Moreover, the risk of all-cause mortality in the high SII group was 1.543 times that of the low SII group (HR = 1.543, 95% CI: 1.017–2.340, *p* = 0.041). Multiple subgroup analyses and sensitivity analyses reverified the stability of these results. ROC curve analysis indicated that SII had a certain predictive value for all-cause mortality (overall population, AUC = 0.605; male, AUC = 0.609; female, AUC = 0.600), and the predictive value of SII was higher than that of platelet-to-lymphocyte ratio (PLR) and monocyte to high-density lipoprotein cholesterol ratio (MHR) (overall population, AUC of PLR = 0.579; AUC of MHR = 0.487). Furthermore, SII combined with NIHSS score and mRS score could improve its predictive value for all-cause mortality in patients with IS (overall population, AUC = 0.683). The Kaplan–Meier survival curve analysis revealed significant differences in the cumulative risk of all-cause mortality among different SII groups, and the cumulative risk of all-cause mortality was higher in the high SII group (*p* < 0.05).

**Conclusion:**

Higher levels of SII were found to be significantly associated with an elevated risk of all-cause mortality in IS patients.

## 1 Introduction

Ischemic stroke (IS) is a major public health problem worldwide, with significant variations in incidence, mortality, and disability rates across different regions and populations. According to the Global Burden of Disease Study, although age-standardized incidence and mortality rates are on a downward trend, the absolute number of IS cases is still increasing due to population aging and the widespread presence of high-risk factors (1). The high mortality risk of IS is closely related to multiple factors. Studies have shown that cardiogenic embolic stroke accounts for approximately one-fourth of all IS, and its severity and disability rate are higher than those of non-cardiogenic strokes (2). In addition, the presence of chronic diseases such as heart disease, diabetes, and hypertension significantly increases the risk of death after stroke (3). In young patients, coronary heart disease is considered a major predictor of new vascular events and death after IS (4). These research results emphasize the importance of managing cardiovascular risk factors after stroke. The risk factors for IS are diverse, including non-modifiable (age, gender, race) and modifiable (hypertension, smoking, diabetes, hyperlipidemia) indicators (5), with established subtype-specific risk profiles in young adults (6). Moreover, recent studies have also revealed the impact of inflammation, environmental pollution and low-density lipoprotein cholesterol (LDL-C) on the incidence of IS (1, 7). The systemic immune-inflammation index (SII), an emerging inflammatory biomarker integrating neutrophil, platelet, and lymphocyte counts, has drawn widespread attention in recent years for its crucial clinical significance in the incidence, progression, risk assessment, and prognosis of stroke (8). Specifically, in a systematic review and meta-analysis, investigators discovered that elevated SII levels were significantly correlated with suboptimal functional outcomes, increased mortality rates, and the incidence of hemorrhagic transformation among stroke patients (9). In the acute IS subgroup, elevated SII levels were independently associated with 90-day poor outcomes in patients receiving intravenous thrombolysis, and SII was significantly positively correlated with the admission National Institutes of Health Stroke Scale (NIHSS) score, suggesting its predictive value for mortality risk (10). Among hospitalized elderly stroke patients (≥ 60 years old, including ischemic and hemorrhagic stroke), non-survivors had significantly higher SII levels than survivors, and multivariate analysis confirmed that SII is an independent predictor of in-hospital all-cause mortality (11). However, in patients with intracerebral hemorrhage (ICH) (*n* = 320), the predictive efficacy of SII did not surpass that of the neutrophil-to-lymphocyte ratio (NLR), and current evidence does not support its use as an independent predictive indicator for mortality risk in ICH patients (12). It should be noted that although several studies [e.g., in populations with asthma, hypertension, acute myocardial infarction (AMI), and cardiorenal metabolic (CKM) syndrome] have shown an association between SII and stroke prevalence or cardiovascular mortality, they did not directly assess the mortality endpoint in stroke patients (13–16). In contrast, studies on the progression of cerebral small vessel disease (CSVD) suggest that SII may be involved in stroke-related pathological processes (e.g., progression of CSVD burden, new cerebral microbleeds) (17, 18), which are indirectly associated with prognosis. Moreover, SII has exhibited high diagnostic and predictive utility in gauging the disease severity of patients with large-artery atherosclerotic stroke (19). Compared to the NLR, SII demonstrated greater diagnostic efficacy (19). Beyond this, SII not only excels in predicting the severity of stroke but also presents independent predictive value in evaluating the risk of stroke-associated pneumonia (SAP). For example, stroke patients with higher SII levels are more likely to develop SAP, indicating that SII can be utilized as an early-detection tool for SAP, which enables clinicians to implement appropriate intervention strategies in a timely manner (20). In addition, the applications of SII in other cardiovascular diseases offer valuable references for its use in the context of stroke. For example, in patients with acute myocardial infarction and peripartum cardiomyopathy, SII has been validated as a key metric for prognosis assessment, and it can effectively predict the risk of in-hospital mortality and the recovery of left ventricular function (21, 22).

However, few studies have systematically compared the discriminatory ability of SII with other inflammatory composite indices such as platelet-to-lymphocyte ratio (PLR) and monocyte to high-density lipoprotein cholesterol ratio (MHR) for all-cause mortality in IS patients, nor have they explored the incremental value of combining SII with core clinical prognostic markers including NIHSS and modified Rankin Scale (mRS) scores. Collectively, these findings underscore that SII, as a readily obtainable blood parameter, plays a pivotal role in the incidence, progression, risk assessment, and prognosis of stroke. Against this backdrop of existing research, the present study endeavored to assess the correlation between SII and all-cause mortality in IS patients, and to determine the optimal cut-off value of SII for characterizing the discriminatory ability for all-cause mortality in this population. The objective was to provide novel insights and a theoretical foundation for the prevention, diagnosis, and treatment strategies targeting inflammation in IS patients, as well as an objective and quantifiable threshold for clinical risk stratification.

## 2 Methods

### 2.1 Study design and participants

**Figure 1:**
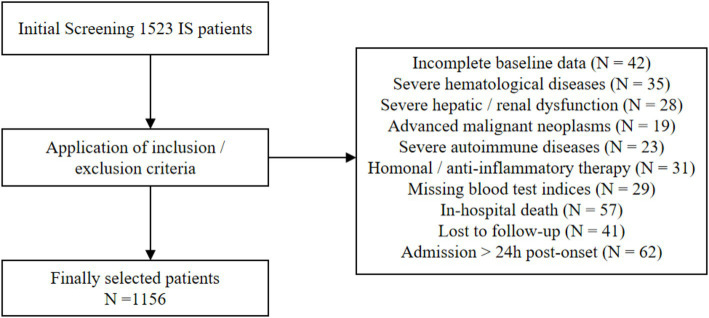
Flow chart of enrollment for the study population of IS. IS, ischemic stroke.

This single-center, retrospective cohort study was carried out at Dandong Central Hospital. The subjects of this study were discharged IS patients. Between January 2024 and December 2024, 1,156 subjects were consecutively recruited according to the following inclusion and exclusion criteria. The relevant flow chart was presented in Figure 1.

Inclusion criteria: (1) Patients diagnosed with IS by computed tomography (CT) or magnetic resonance imaging (MRI); (2) Patients with complete baseline clinical characteristics, demographic data, and laboratory test results; (3) Patients admitted within 24 h of symptom onset.

Exclusion criteria: (1) Patients with incomplete baseline data; (2) Patients admitted more than 24 h after the onset of symptoms; (3) Patients with severe hematological diseases; (4) Patients with severe hepatic or renal dysfunction; (5) Patients with advanced malignant tumors; (6) Patients with severe autoimmune diseases; (7) Patients receiving hormone or anti-inflammatory treatment; (8) Patients with missing blood test indices; (9) Patients who died during hospitalization; (10) Patients lost to follow-up. This study protocol was approved by the Ethics Committee of Dandong Central Hospital (Approval No. DDSZXYY-2025-48) and was conducted in accordance with the principles of the Declaration of Helsinki. As this was a retrospective study and all data were analyzed anonymously, the requirement for written informed consent from patients was reviewed and waived by the Ethics Committee of Dandong Central Hospital.

### 2.2 Data collection and variable definitions

Within the electronic medical record system of our hospital, data encompassing demographics, anthropometrics, comorbidities, prior medication history, and blood biomarkers were collected for all patients included in this study. Demographic data incorporated patient age, sex, smoking, and drinking status. Smoking was defined as a history of regular smoking or current active smoking (regardless of subsequent cessation). Drinking was defined as a history of regular alcohol intake or ongoing consumption (regardless of abstinence attempts). Anthropometric measurements included height, weight, body mass index (BMI), systolic blood pressure (SBP), and diastolic blood pressure (DBP). BMI was calculated as weight (in kilograms) divided by the square of height (in meters).

Comorbid conditions included hypertension, diabetes, hyperlipidemia, previous cerebral infarction, and the duration of cerebral infarction. Hypertension was defined as a well-established prior diagnosis of hypertension, an SBP of ≥ 140 mmHg or a DBP of ≥ 90 mmHg during the current hospitalization, or ongoing antihypertensive medication treatment (23). Diabetes was defined as a definite prior diagnosis of diabetes, a fasting blood glucose (FBG) level of ≥ 7.0 mmol/L, a glycated hemoglobin (HbA1c) level of ≥ 6.5%, current receipt of antidiabetic medication, or a blood glucose level of ≥ 11.1 mmol/L detected 2 h after an oral glucose tolerance test (24). Hyperlipidemia was defined as a definite prior diagnosis of hyperlipidemia, a triglyceride level of ≥ 2.3 mmol/L, a total cholesterol (TC) level of ≥ 6.2 mmol/L, or a LDL-C level of ≥ 4.1 mmol/L during the current hospitalization (25). A history of cerebral infarction was defined as a documented prior diagnosis of cerebral infarction. The duration of cerebral infarction was recorded as the time elapsed from the initial onset of cerebral infarction symptoms to the current hospitalization. Prior medication history involved information on patients’ previous use of antihypertensive drugs, antidiabetic agents, lipid-lowering medications, anti-platelet drugs, among others. Blood biomarker data included white blood cell count (WBC), neutrophil count, lymphocyte count, monocyte count, hemoglobin concentration, platelet count, FBG, uric acid, estimated glomerular filtration rate (eGFR), homocysteine, triglycerides, TC, LDL-C, and high-density lipoprotein cholesterol (HDL-C). Additionally, we calculated two other inflammatory composite indices for comparative analysis: PLR, defined as platelet count divided by lymphocyte count; and MHR, defined as monocyte count divided by HDL-C level, with all cell count units expressed as ×10^9^/L. All these biomarkers were obtained through a standardized procedure. Specifically, trained nursing staff in our hospital collected venous blood samples from the antecubital veins of patients who had fasted for at least 8 h. All blood samples were derived from the patients’ first routine hematological tests after admission (with fasting time ≥ 8 h), which were generally completed within 24 h of hospital admission. From a clinical background and practical perspective, the patients included in this study were mainly in the acute or subacute phase of IS, as they were admitted within 24 h of symptom onset. These samples were then promptly transferred to the central laboratory of our hospital, where laboratory technicians conducted measurements following established protocols. Baseline stroke severity was assessed using the NIHSS score, which was evaluated by certified neurologists at admission. Pre-stroke residual functional impairment was assessed using the mRS score, which was collected through medical record review and patient/family interview to reflect the functional status before the index stroke onset. Both NIHSS score and pre-stroke mRS score were fully collected, verified for data integrity, and included as key covariates in all statistical analyses.

### 2.3 Definition and grouping of systemic immune-inflammation index

The SII is calculated using peripheral blood cell counts and serves to reflect the integrated state of systemic immunity and inflammation. The formula for SII is as follows: SII = (neutrophil count × platelet count)/lymphocyte count (26). Herein, all cell count units are expressed as ×10^9^/L. Two different thresholds were used to stratify the study subjects into high and low SII groups: (1) Median-based grouping: The median value of SII in the study population (620.81) was used for grouping, with low SII group (SII ≤ 620.81, *n* = 578) and high SII group (SII > 620.81, *n* = 578), to ensure approximately equal sample size between the two groups and avoid selection bias caused by significant sample size disparity. (2) Optimal cut-off value-based grouping: The optimal cut-off value of SII for characterizing the discriminatory ability for all-cause mortality in IS patients was determined by the maximum Youden index (Youden index = sensitivity + specificity − 1) derived from the ROC curve. It should be noted that this cut-off value was obtained through exploratory analysis, and the ROC curve was not used to establish a clinical predictive model. The optimal cut-off value was determined to be 986.83, with low SII group (SII ≤ 986.83, *n* = 851) and high SII group (SII > 986.83, *n* = 305).

### 2.4 Follow-up and outcome indicators

The starting point of the follow-up was the date of the patient’s discharge. The ending point of the follow-up was determined as the time of all-cause mortality or September 2025, with precedence given to the earlier event. We defined all-cause mortality during the follow-up period with the follow-up endpoint set from patient discharge to September 2025, and this was primarily based on the following considerations. First, the present study focused on the long-term mortality risk of stroke patients after discharge, which is more closely aligned with clinical follow-up practices and the prognostic assessment of IS patients in clinical settings. Second, in this study, we had already excluded patients who died during hospitalization. Therefore, if the follow-up were to start from the onset of stroke, it would inevitably include patients who died in hospital, making it impossible to analyze the prognostic findings related to long- and medium-term mortality in out-of-hospital settings. Third, our choice of using the discharge date as the starting point for follow-up is also consistent with the approach reported in the majority of relevant studies. The follow-up strategy involved multiple approaches. First, we meticulously examined the outpatient and emergency department records, as well as the inpatient medical files, of patients who had made several visits to our hospital after being discharged. Additionally, telephone follow-up was carried out. Through these telephone contacts, we gathered follow-up information regarding the patients’ post-discharge status from either the patients themselves or their family members. The key outcome measure in this study was all-cause mortality, which was defined as death triggered by any disease or incident. Based on the occurrence of all-cause mortality, the study cohort was partitioned into two groups: the all-cause mortality group (*n* = 97) and the group with non-all-cause mortality (*n* = 1,059).

### 2.5 Statistical analysis

All statistical analyses were conducted using SPSS 27.0 software (IBM Corporation, Armonk, New York, USA). Categorical variables were presented as frequencies (percentages). The chi-square test or Fisher’s exact test was employed to determine the differences between two groups. For all continuous variables, normality was examined using the Shapiro–Wilk test. Given that all continuous variables did not conform to a normal distribution, they were expressed as the median (interquartile range), and the Mann–Whitney U test was utilized to assess the differences between groups. For the handling of missing data, this study was a retrospective analysis based on the hospital electronic medical record system. During participant enrollment, we excluded patients with missing parameters required for SII calculation (including neutrophil count, platelet count, and lymphocyte count) and those lost to follow-up. We ensured no missing data for the independent and dependent variables, and complete data were also available for demographic characteristics, anthropometric measures, comorbidities, and medication history. However, a small number of individual hematological indicators had minor missing values, with a missing rate of less than 5% for each variable. We therefore used mean imputation, a conventional simple imputation method commonly adopted in clinical research, to address these limited missing data. In summary, the vast majority of variables in this study had no missing values, and the few remaining missing data were imputed using the clinically common mean imputation approach. Univariate Cox regression analysis was carried out to evaluate the correlation of each variable with all-cause mortality. Subsequently, variables with a *p* value less than 0.05 were selected to establish three multivariate Cox regression models with incremental adjustment for confounding factors: Model 1: Adjusted for age, baseline NIHSS score, and pre-stroke mRS score; Model 2: Further adjusted for hyperlipidemia, a history of previous cerebral infarction, lipid-lowering medications, and anti-platelet drugs on the basis of Model 1; Model 3 (fully adjusted model): Additionally adjusted for SBP, DBP, FBG, uric acid, and eGFR on the basis of Model 2. The significant association between SII and all-cause mortality was further evaluated within these three multivariate Cox regression models. Next, ten subgroups were formed based on five variables: age, smoking status, drinking, diabetes, and a history of previous cerebral infarction. The stratified association between SII and all-cause mortality was re-evaluated in the fully adjusted model. In the sensitivity analysis, patients without hypertension and those without hyperlipidemia were separately excluded. The correlation between SII and all-cause mortality was then re-verified among these subsets of patients. Descriptive and exploratory receiver operating characteristic (ROC) curve analysis was performed for the following purposes: (1) To assess and compare the discriminatory ability of SII, PLR and MHR for all-cause mortality in the overall population, male subgroup and female subgroup; (2) To evaluate the incremental discriminatory value of combining SII with NIHSS score, mRS score, or both for all-cause mortality. The area under the curve (AUC) with 95% CI was calculated for each ROC curve. We explicitly state that the ROC curve and AUC values were not applied for the development or validation of a formal clinical predictive model, and only served to descriptively characterize the discriminatory performance of biomarkers for the study endpoint. Finally, the Kaplan–Meier survival curve with log-rank test was applied to evaluate the differences in the cumulative risk of all-cause mortality among different SII groups. All statistical tests were two-tailed, and a *p* value less than 0.05 was considered to indicate a statistically significant difference.

## 3 Results

### 3.1 Baseline characteristics grouped by the median of SII

The baseline characteristics grouped by the median of SII were presented in Table 1. The total sample size was 1,156 individuals. The median age was 70 years (interquartile range: 64.00–77.00), among which 645 were male, accounting for 55.8% of the total population. Based on the median of SII, the subjects were divided into two groups: the low SII group (*n* = 578) and the high SII group (*n* = 578). When compared with the low SII group, the high SII group exhibited a higher prevalence of antihypertensive drugs use, higher levels of BMI, WBC, neutrophil count, platelet count (*p* < 0.05). Conversely, the high SII group had a lower prevalence of smoking history, a shorter duration of cerebral infarction, and lower lymphocyte counts (all *p* < 0.05). Notably, the high SII group had a significantly higher pre-stroke mRS score than the low SII group (*p* = 0.010), while there was no statistically significant difference in baseline NIHSS score between the two groups (*p* = 0.180), the high SII group also had a significantly higher proportion of all-cause mortality (10.7% vs. 6.1%, *p* = 0.004). However, other variables such as age, sex, drinking, hypertension, diabetes, hyperlipidemia, and previous history of cerebral infarction did not show statistically significant differences between the two groups (*p* > 0.05).

**Table 1:** Baseline characteristics grouped by median of SII.

Variables	Total population	Low SII	High SII	*p* value
*N*	1,156	578	578	
Age, years	70.00 (64.00, 77.00)	70.00 (63.00, 76.00)	71.00 (64.00, 78.00)	0.082
Sex, *n* (%)				0.214
Male	645 (55.8%)	333 (57.6%)	312 (54.0%)	
Female	511 (44.2%)	245 (42.4%)	266 (46.0%)	
Smoking, *n* (%)	435 (37.6%)	237 (41.0%)	198 (34.3%)	0.018
Drinking, *n* (%)	342 (29.6%)	183 (31.7%)	159 (27.5%)	0.122
Hypertension, *n* (%)	1,048 (90.7%)	518 (89.6%)	530 (91.7%)	0.225
Diabetes, *n* (%)	521 (45.1%)	250 (43.3%)	271 (46.9%)	0.214
Hyperlipidemia, *n* (%)	1,070 (92.6%)	536 (92.7%)	534 (92.4%)	0.823
Previous cerebral infarction, *n* (%)	626 (54.2%)	301 (52.1%)	325 (56.2%)	0.157
Course of cerebral infarction, day	1.00 (0.19, 3.38)	2.00 (0.25, 5.00)	1.00 (0.19, 3.00)	<0.001
Antihypertensive drugs, *n* (%)	821 (71.0%)	393 (68.0%)	428 (74.0%)	0.023
Antidiabetic drugs, *n* (%)	420 (36.3%)	200 (34.6%)	220 (38.1%)	0.221
Lipid-lowering drugs, *n* (%)	1,066 (92.2%)	534 (92.4%)	532 (92.0%)	0.826
Antiplatelet drugs, *n* (%)	1,041 (90.1%)	525 (90.8%)	516 (89.3%)	0.376
BMI, kg/m^2^	24.54 (22.49, 27.06)	24.49 (22.49, 26.99)	24.69 (22.49, 27.34)	<0.001
SBP, mmHg	151.00 (139.00, 166.00)	150.00 (138.00, 163.00)	151.00 (138.00, 166.00)	0.202
DBP, mmHg	84.00 (78.00, 91.00)	84.00 (77.00, 90.00)	85.00 (78.00, 91.00)	0.184
WBC, x10^9^/L	6.93 (5.73, 8.66)	6.12 (5.21, 7.28)	7.87 (6.63, 9.55)	<0.001
Neutrophil count, x10^9^/L	4.78 (3.71, 6.23)	3.79 (3.11, 4.62)	6.01 (4.90, 7.75)	<0.001
Lymphocyte count, x10^9^/L	1.48 (1.08, 1.89)	1.74 (1.39, 2.21)	1.19 (0.92, 1.61)	<0.001
Monocyte count, x10^9^/L	0.37 (0.29, 0.48)	0.37 (0.30, 0.46)	0.38 (0.29, 0.49)	0.429
Hemoglobin, g/L	138.00 (127.00, 149.00)	139.00 (128.00, 150.00)	137.00 (126.00, 148.00)	0.053
Platelet count, x10^9^/L	198.00 (166.00, 238.00)	188.00 (155.00, 216.00)	218.00 (181.00, 255.00)	<0.001
FBG, mmol/L	5.70 (5.00, 7.30)	5.60 (5.00, 7.18)	5.74 (5.00, 7.43)	0.417
Uric acid, μmol/L	325.00 (266.00, 389.75)	320.00 (266.00, 381.75)	335.00 (265.00, 400.00)	0.223
eGFR, mL/min/1.73m^2^	101.49 (80.64, 122.41)	102.70 (85.29, 121.16)	99.85 (77.01, 123.96)	0.090
Homocysteine, μmol/L	12.60 (10.50, 15.80)	12.60 (10.40, 15.60)	12.80 (10.50, 15.80)	0.279
Triglycerides, mmo/L	1.34 (0.97, 1.91)	1.36 (1.00, 1.92)	1.33 (0.97, 1.85)	0.052
Total cholesterol, mmo/L	4.43 (3.65, 5.23)	4.43 (3.58, 5.15)	4.42 (3.69, 5.29)	0.693
LDL-C, mmol/L	2.71 (2.10, 3.32)	2.70 (2.08, 3.28)	2.73 (2.12, 3.35)	0.792
HDL-C, mmol/L	1.13 (0.95, 1.32)	1.11 (0.95, 1.33)	1.14 (0.86, 1.32)	0.424
mRS score	3.00 (1.00, 4.00)	2.00 (1.00, 3.00)	3.00 (1.00, 4.00)	0.010
NIHSS score	2.00 (1.00, 4.00)	2.00 (1.00, 4.00)	2.00 (1.00, 4.00)	0.180
All-cause mortality, *n* (%)				0.004
Yes	97 (8.4%)	35 (6.1%)	62 (10.7%)	
No	1,059 (91.6%)	543 (93.9%)	516 (89.3%)	

SII, Systemic-immune inflammation index; BMI, Body mass index; SBP, Systolic blood pressure; DBP, Diastolic blood pressure; WBC, White blood cell count; FBG, Fasting blood glucose; eGFR, Estimated glomerular filtration rate; LDL-C, Low-density lipoprotein cholesterol; HDL-C, High-density lipoprotein cholesterol; mRS, modified Rankin Scale; NIHSS, National Institutes of Health Stroke Scale.

### 3.2 Baseline characteristics grouped by all-cause mortality

As presented in Table 2, subjects were categorized into two groups according to the occurrence of all-cause mortality: the non-all-cause mortality group (*n* = 1,059) and the all-cause mortality group (*n* = 97). In comparison with the non-all-cause mortality group, the all-cause mortality group demonstrated a higher age, a greater proportion of previous cerebral infarction, and elevated levels of SBP, DBP, WBC, neutrophil count, FBG, uric acid, and homocysteine (*p* < 0.05). Conversely, the all-cause mortality group had a lower proportion of hyperlipidemia, a shorter duration of cerebral infarction, a lower utilization rate of lipid-lowering drugs and anti-platelet drugs, as well as reduced levels of lymphocyte count, hemoglobin, and eGFR (*p* < 0.05). The all-cause mortality group had significantly higher baseline NIHSS scores (*p* < 0.001) and pre-stroke mRS scores (*p* < 0.001) than the non-all-cause mortality group. More significantly, the level of SII was markedly higher in the all-cause mortality group (*p* < 0.001). Nevertheless, other variables including sex, smoking status, drinking, hypertension, and diabetes did not exhibit statistically significant differences between the two groups (*p* > 0.05).

**Table 2:** Baseline characteristics grouped by all-cause mortality.

Variables	Non all-cause mortality	All-cause mortality	*p* value
*N*	1,059	97	
Age, years	70.00 (63.00, 76.00)	76.00 (70.00, 83.00)	<0.001
Sex, *n* (%)			0.505
Male	594 (56.1%)	51 (52.6%)	
Female	465 (43.9%)	46 (47.4%)	
Smoking, *n* (%)	401 (37.9%)	34 (35.1%)	0.584
Drinking, *n* (%)	312 (29.5%)	30 (30.9%)	0.762
Hypertension, *n* (%)	957 (90.4%)	91 (93.8%)	0.264
Diabetes, *n* (%)	473 (44.7%)	48 (49.5%)	0.361
Hyperlipidemia, *n* (%)	989 (93.4%)	81 (83.5%)	<0.001
Previous cerebral infarction, *n* (%)	561 (53.0%)	65 (67.0%)	0.008
Course of cerebral infarction, days	1.00 (0.21, 4.00)	1.00 (0.13, 2.50)	0.021
Antihypertensive drugs, *n* (%)	751 (70.9%)	70 (72.2%)	0.795
Antidiabetic drugs, *n* (%)	384 (36.3%)	36 (37.1%)	0.867
Lipid-lowering drugs, *n* (%)	986 (93.1%)	80 (82.5%)	<0.001
Antiplatelet drugs, *n* (%)	971 (91.7%)	70 (72.2%)	<0.001
BMI, kg/m^2^	24.60 (22.51, 27.04)	23.44 (21.48, 28.55)	0.371
SBP, mmHg	151.00 (139.00, 165.00)	151.00 (140.00, 172.00)	0.031
DBP, mmHg	84.00 (78.00, 90.00)	89.00 (74.00, 97.00)	0.042
WBC, ×10^9^/L	6.79 (5.70, 8.33)	7.68 (5.48, 9.99)	<0.001
Neutrophil count, ×10^9^/L	4.69 (3.68, 5.93)	5.77 (3.65, 7.98)	<0.001
Lymphocyte count, ×10^9^/L	1.50 (1.11, 1.89)	1.31 (0.96, 1.72)	0.002
Monocyte count, ×10^9^/L	0.37 (0.29, 0.46)	0.39 (0.29, 0.41)	0.124
Hemoglobin, g/L	139.00 (128.00, 149.00)	128.00 (113.00, 143.00)	<0.001
Platelet count, ×10^9^/L	199.00 (168.00, 235.00)	200.00 (151.00, 245.00)	0.496
FBG, mmol/L	5.61 (5.00, 7.17)	6.30 (5.40, 8.59)	0.001
Uric acid, μmol/L	324.50 (265.25, 385.00)	363.00 (274.00, 454.00)	0.024
eGFR, mL/min/1.73m^2^	101.21 (82.27, 121.86)	88.77 (52.73, 115.59)	<0.001
Homocysteine, μmol/L	12.50 (10.40, 15.50)	14.90 (11.81, 17.70)	0.003
Triglycerides, mmol/L	1.34 (0.99, 1.87)	1.32 (0.96, 1.97)	0.465
Total cholesterol, mmo/L	4.42 (3.63, 5.18)	4.74 (4.00, 5.53)	0.266
LDL-C, mmol/L	2.70 (2.09, 3.28)	3.04 (2.53, 3.70)	0.199
HDL-C, mmol/L	1.13 (0.95, 1.33)	1.14 (1.03, 1.25)	0.825
mRS score	3.00 (1.00, 4.00)	3.00 (2.00, 4.00)	<0.001
NIHSS score	2.00 (1.00, 4.00)	3.00 (1.50, 7.50)	<0.001
SII	609.82 (418.06, 945.71)	726.87 (360.21, 1457.99)	<0.001
Log_10_SII	2.79 (2.62, 2.98)	2.86 (2.56, 3.16)	<0.001
Standardized SII	−0.33 (−0.54, 0.02)	−0.21 (−0.60, 0.56)	<0.001
SII (Median), *n* (%)			0.004
Low SII	543 (51.3%)	35 (36.1%)	
High SII	516 (48.7%)	62 (63.9%)	
SII (Optimal cut-off value), *n* (%)			<0.001
Low SII	801 (75.6%)	50 (51.5%)	
High SII	258 (24.4%)	47 (48.5%)	

BMI, Body mass index; SBP, Systolic blood pressure; DBP, Diastolic blood pressure; WBC, White blood cell count; FBG, Fasting blood glucose; eGFR, Estimated glomerular filtration rate; LDL-C, Low-density lipoprotein cholesterol; HDL-C, High-density lipoprotein cholesterol; SII, Systemic-immune inflammation index; mRS, modified Rankin Scale; NIHSS, National Institutes of Health Stroke Scale.

### 3.3 Univariate Cox regression analysis of all-cause mortality

As presented in Table 3, the univariate Cox regression analysis revealed that age, hyperlipidemia, a history of previous cerebral infarction, lipid-lowering drugs, anti-platelet drugs, SBP, DBP, WBC, neutrophil count, lymphocyte count, monocyte count, hemoglobin, FBG, uric acid, eGFR, pre-stroke mRS score, baseline NIHSS score, and SII were all significantly associated with the risk of all-cause mortality (*p* < 0.05). Specifically, for every one-standard deviation (SD) increase in SII, the risk of all-cause mortality increased by 23.1% (HR = 1.231, 95% CI: 1.090–1.391, *p* < 0.001). Moreover, the risk of all-cause mortality in the high SII group (median-based grouping) was 1.829 times that in the low SII group (HR = 1.829, 95% CI: 1.209–2.768, *p* = 0.004). For the optimal cut-off value-based grouping, the high SII group had a 2.765-fold higher risk of all-cause mortality than the low SII group (HR = 2.765, 95% CI: 1.856–4.118, *p* < 0.001). For the key clinical markers of stroke severity, per 1-point increase in pre-stroke mRS score was associated with a 43.3% increase in all-cause mortality risk (HR = 1.433, 95% CI: 1.223–1.679, *p* < 0.001), and per 1-point increase in baseline NIHSS score was associated with a 10.3% increase in all-cause mortality risk (HR = 1.103, 95% CI: 1.064–1.144, *p* < 0.001). However, other variables such as sex, smoking status, drinking, hypertension, and diabetes did not show a significant association with all-cause mortality (*p* > 0.05).

**Table 3:** Univariate Cox regression analysis of all-cause mortality risk.

Variables	HR	95% CI	*p* value
Age	1.089	1.066, 1.113	<0.001
Male	0.876	0.588, 1.305	0.516
Smoking	0.890	0.587, 1.351	0.585
Drinking	1.067	0.693, 1.641	0.769
Hypertension	1.577	0.690, 3.602	0.280
Diabetes	1.210	0.813, 1.802	0.347
Hyperlipidemia	0.395	0.231, 0.675	0.001
Previous cerebral infarction	1.743	1.142, 2.662	0.010
Course of cerebral infarction	0.973	0.942, 1.005	0.097
Antihypertensive drugs	1.052	0.675, 1.640	0.823
Antidiabetic drugs	1.032	0.684, 1.558	0.881
Lipid-lowering drugs	0.378	0.224, 0.638	<0.001
Antiplatelet drugs	0.263	0.169, 0.411	<0.001
BMI	0.973	0.918, 1.030	0.347
SBP	1.089	1.066, 1.113	0.003
DBP	1.023	1.006, 1.040	0.008
WBC	1.124	1.076, 1.173	<0.001
Neutrophil count	1.136	1.088, 1.185	<0.001
Lymphocyte count	0.663	0.474, 0.927	0.016
Monocyte count	3.366	1.065, 10.632	0.039
Hemoglobin	0.975	0.967, 0.983	<0.001
Platelet count	0.999	0.996, 1.002	0.502
FBG	1.113	1.062, 1.167	<0.001
Uric acid	1.002	1.001, 1.004	0.004
eGFR	0.988	0.982, 0.994	<0.001
Homocysteine	1.008	0.993, 1.022	0.301
Triglycerides	0.930	0.772, 1.120	0.442
Total cholesterol	1.086	0.913, 1.292	0.350
LDL-C	1.133	0.910, 1.409	0.265
HDL-C	0.857	0.418, 1.757	0.673
mRS score	1.433	1.223, 1.679	<0.001
NIHSS score	1.103	1.064, 1.144	<0.001
SII	1.000	1.000, 1.000	<0.001
Log_10_SII	2.832	1.598, 5.021	<0.001
Standardized SII	1.231	1.090, 1.391	<0.001
SII (Median), *n* (%)			0.004
Low SII	0.547	0.361, 0.827	
High SII	1.829	1.209, 2.768	
SII (Optimal cut-off value), *n* (%)			<0.001
Low SII	0.362	0.243, 0.589	
High SII	2.765	1.856, 4.118	

BMI, Body mass index; SBP, Systolic blood pressure; DBP, Diastolic blood pressure; WBC, White blood cell count; FBG, Fasting blood glucose; eGFR, Estimated glomerular filtration rate; LDL-C, Low-density lipoprotein cholesterol; HDL-C, High-density lipoprotein cholesterol; SII, Systemic-immune inflammation index; mRS, modified Rankin Scale; NIHSS, National Institutes of Health Stroke Scale.

### 3.4 Multivariate Cox regression analysis of the SII and all-cause mortality

As presented in Table 4, three multivariate Cox regression models with incremental adjustment for confounding factors were constructed to verify the independent association between SII and all-cause mortality, with results detailed as follows: In Model 1 (adjusted for age, pre-stroke mRS score, and baseline NIHSS score), a one-SD increase in SII was associated with a 17.2% elevation in the risk of all-cause mortality (HR = 1.172, 95% CI: 1.021–1.346, *p* = 0.024). Moreover, the risk of all-cause mortality in the high SII group (median-based) was 1.560 times that of the low SII group (HR = 1.560, 95% CI: 1.028–2.366, *p* = 0.036). Notably, the optimal cut-off value-based high SII group showed a more pronounced elevation in mortality risk, with a 2.196-fold higher risk than the low SII group (HR = 2.196, 95% CI: 1.468–3.285, *p* < 0.001); In Model 2 (additionally adjusted for hyperlipidemia, a history of previous cerebral infarction, lipid-lowering medications, and anti-platelet drugs), a one-SD increment in SII was associated with a 15.9% increase in the risk of all-cause mortality (HR = 1.159, 95% CI: 1.006–1.335, *p* = 0.041). The risk of all-cause mortality in the high SII group (median-based) was 1.551 times that of the low SII group (HR = 1.551, 95% CI: 1.024–2.352, *p* = 0.038). For the optimal cut-off value-based grouping, the high SII group still had a 2.108-fold higher risk of all-cause mortality (HR = 2.108, 95% CI: 1.406–3.160, *p* < 0.001), with the statistical significance remaining at the p < 0.001 level; In the fully adjusted Model 3 (additionally adjusted for SBP, DBP, FBG, uric acid, and eGFR on the basis of Model 2), a one-SD increase in SII was associated with a 17.2% rise in the risk of all-cause mortality (HR = 1.172, 95% CI: 1.019–1.348, *p* = 0.026). The risk of all-cause mortality in the high SII group (median-based) was 1.543 times that of the low SII group (HR = 1.543, 95% CI: 1.017–2.340, *p* = 0.041). Most importantly, the optimal cut-off value of 986.83 showed robust and stronger prognostic discrimination: patients with SII > 986.83 had a 2.156-fold increased risk of all-cause mortality compared with those with SII ≤ 986.83 (HR = 2.156, 95% CI: 1.440–3.230, *p* < 0.001), even after full adjustment for all confounding factors. The HR value of the optimal cut-off value-based grouping was 39.7% higher than that of the median-based grouping.

**Table 4:** Multivariate Cox regression analysis of SII and all-cause mortality.

Variables	Model 1			Model 2			Model 3		
	HR	95% CI	*p* value	HR	95% CI	*p* value	HR	95% CI	*p* value
SII (continuous variable)									
SII	1.000	1.000, 1.000	0.024	1.000	1.000, 1.000	0.041	1.000	1.000, 1.000	0.026
Log_10_SII	2.042	1.168, 3.569	0.012	1.930	1.110, 3.356	0.020	1.975	1.113, 3.504	0.020
Standardized SII	1.172	1.021, 1.346	0.024	1.159	1.006, 1.335	0.041	1.172	1.019, 1.348	0.026
SII (Median)									
Low SII	Ref			Ref			Ref		
High SII	1.560	1.028, 2.366	0.036	1.551	1.024, 2.352	0.038	1.543	1.017, 2.340	0.041
SII (Optimal cut-off value)									
Low SII	Ref			Ref			Ref		
High SII	2.196	1.468, 3.285	<0.001	2.108	1.406, 3.160	<0.001	2.156	1.440, 3.230	<0.001

Model 1: adjusted for age, mRS score, NIHSS score; Model 2: adjusted for age, mRS score, NIHSS score, hyperlipidemia, previous cerebral infarction, lipid-lowering drugs, and antiplatelet drugs; Model 3: adjusted for age, mRS score, NIHSS score, hyperlipidemia, previous cerebral infarction, lipid-lowering drugs, antiplatelet drugs, SBP, DBP, FBG, uric acid, and eGFR.

SII, Systemic-immune inflammation index; SBP, Systolic blood pressure; DBP, Diastolic blood pressure; FBG, Fasting blood glucose; eGFR, Estimated glomerular filtration rate; mRS, modified Rankin Scale; NIHSS, National Institutes of Health Stroke Scale.

### 3.5 Subgroup analysis of the SII and all-cause mortality

As presented in Table 5, subgroup analysis was conducted within the fully adjusted model to explore the stratified association between SII and all-cause mortality across different clinical subgroups, with results detailed as follows. In the subgroup of patients aged 70 years or older, the risk of all-cause mortality in the high SII group (median-based) was 1.623-fold that of the low SII group (HR = 1.623, 95% CI: 1.017–2.590, *p* = 0.042). Moreover, for every one-SD increase in SII, the risk of all-cause mortality increased by 16.9% (HR = 1.169, 95% CI: 1.004–1.362, *p* = 0.044). In the subgroup of smokers, a one-SD increase in SII was associated with a 36.5% increase in the risk of all-cause mortality (HR = 1.365, 95% CI: 1.081–1.723, *p* = 0.009). In the subgroup with a history of previous cerebral infarction, the risk of all-cause mortality in the high SII group (median-based) was 1.782 times that of the low SII group (HR = 1.782, 95% CI: 1.063–2.986, *p* = 0.028). For the optimal cut-off value-based SII grouping, the prognostic performance was more prominent and stable across all key subgroups, with higher HR values and more significant statistical differences. In patients aged ≥ 70 years, the high SII group defined by the optimal cut-off value had a 2.168-fold higher risk of all-cause mortality (HR = 2.168, 95% CI: 1.386–3.390, *p* < 0.001). In smokers, the optimal cut-off value-based high SII group had a 3.854-fold increased mortality risk (HR = 3.854, 95% CI: 1.923–7.721, p < 0.001), showing an extremely strong prognostic association. In patients with previous cerebral infarction, the optimal cut-off value-based high SII group had a 2.175-fold higher risk of all-cause mortality (HR = 2.175, 95% CI: 1.327–3.566, *p* = 0.002). In addition, the optimal cut-off value of SII also showed significant prognostic value in several subgroups where the median-based grouping did not reach statistical significance, including non-smokers (HR = 1.799, 95% CI: 1.072–3.017, *p* = 0.026), patients with drinking (HR = 2.755, 95% CI: 1.320–5.752, *p* = 0.007), patients without drinking (HR = 1.987, 95% CI: 1.213–3.254, *p* = 0.006), patients with diabetes (HR = 2.214, 95% CI: 1.246–3.934, p = 0.007), and patients without diabetes (HR = 1.990, 95% CI: 1.119–3.539, *p* = 0.019).

**Table 5:** Subgroup analysis of the correlation between SII and all-cause mortality.

Subgroups	High SII vs. Low SII (Median)			Standardized SII			High SII vs. Low SII (Optimal cut-off value)		
	HR	95% CI	*p* value	HR	95% CI	*p* value	HR	95% CI	*p* value
Age									
< 70 years	1.015	0.370, 2.783	0.977	1.151	0.783, 1.692	0.474	2.163	0.761, 6.153	0.148
≥ 70 years	1.623	1.017, 2.590	0.042	1.169	1.004, 1.362	0.044	2.168	1.386, 3.390	<0.001
Smoking									
Yes	2.101	0.959, 4.602	0.063	1.365	1.081, 1.723	0.009	3.854	1.923, 7.721	<0.001
No	1.181	0.703, 1.984	0.529	1.062	0.875, 1.290	0.541	1.509	0.888, 2.564	0.128
Drinking									
Yes	1.569	0.694, 3.550	0.279	1.239	0.900, 1.707	0.188	2.755	1.320, 5.752	0.007
No	1.474	0.886, 2.453	0.135	1.133	0.956, 1.343	0.151	1.987	1.213, 3.254	0.006
Diabetes									
Yes	1.860	0.976, 3.576	0.059	1.220	0.961, 1.550	0.102	2.214	1.246, 3.934	0.007
No	1.085	0.600, 1.961	0.788	1.060	0.853, 1.317	0.598	1.990	1.119, 3.539	0.019
Previous cerebral infarction									
Yes	1.782	1.063, 2.986	0.028	1.156	0.976, 1.369	0.093	2.175	1.327, 3.566	0.002
No	0.957	0.448, 2.044	0.909	1.208	0.899, 1.624	0.210	2.115	1.022, 4.376	0.043

Subgroup analysis adjusted for age, mRS score, NIHSS score, hyperlipidemia, previous cerebral infarction, lipid-lowering drugs, antiplatelet drugs, SBP, DBP, FBG, uric acid, and eGFR.

SII, Systemic-immune inflammation index; SBP, Systolic blood pressure; DBP, Diastolic blood pressure; FBG, Fasting blood glucose; eGFR, Estimated glomerular filtration rate; mRS, modified Rankin Scale; NIHSS, National Institutes of Health Stroke Scale.

### 3.6 Sensitivity analysis of the association between SII and all-cause mortality

As presented in Table 6, sensitivity analysis was performed by separately excluding patients without hypertension and those without hyperlipidemia to re-verify the stability of the association between SII and all-cause mortality. When patients without hypertension were excluded, the multivariate Cox regression analysis demonstrated that in the partially adjusted Model 1 and Model 2, higher levels of SII remained significantly associated with the risk of all-cause mortality (all *p* < 0.05). In the fully adjusted Model 3, for every one-SD increase in SII, the risk of all-cause mortality increased by 17.0% (HR = 1.170, 95% CI: 1.016–1.347, *p* = 0.029). Moreover, the risk of all-cause mortality in the high SII group (median-based) was 1.603 times that of the low SII group (HR = 1.603, 95% CI: 1.040–2.469, *p* = 0.032). For the optimal cut-off value-based grouping, the high SII group still had a 2.220-fold higher risk of all-cause mortality (HR = 2.220, 95% CI: 1.462–3.371, *p* < 0.001) in the fully adjusted model.

**Table 6:** Multivariate Cox regression analysis of SII and all-cause mortality: exclude patients without hypertension or hyperlipidemia.

Variables	Model 1			Model 2			Model 3		
	HR	95% CI	*p* value	HR	95% CI	*p* value	HR	95% CI	*p* value
With hypertension									
SII (Continuous variable)									
SII	1.000	1.000, 1.000	0.029	1.000	1.000, 1.000	0.031	1.000	1.000, 1.000	0.029
Log_10_SII	2.129	1.202, 3.769	0.010	2.137	1.220, 3.743	0.008	2.074	1.152, 3.733	0.015
Standardized SII	1.168	1.016, 1.343	0.029	1.174	1.015, 1.358	0.031	1.170	1.016, 1.347	0.029
SII (Median)									
Low SII	Ref			Ref			Ref		
High SII	1.634	1.061, 2.517	0.026	1.603	1.041, 2.469	0.032	1.603	1.040, 2.469	0.032
SII (Optimal cut-off value)									
Low SII	Ref			Ref			Ref		
High SII	2.254	1.488, 3.414	<0.001	2.071	1.362, 3.150	<0.001	2.220	1.462, 3.371	<0.001
With hyperlipidemia									
SII (Continuous variable)									
SII	1.000	1.000, 1.000	0.004	1.000	1.000, 1.000	0.006	1.000	1.000, 1.000	0.008
Log_10_SII	2.537	1.375, 4.680	0.003	2.430	1.313, 4.496	0.005	2.324	1.228, 4.398	0.010
Standardized SII	1.217	1.063, 1.392	0.004	1.212	1.056, 1.393	0.006	1.209	1.051, 1.390	0.008
SII (Median)									
Low SII	Ref			Ref			Ref		
High SII	1.788	1.122, 2.849	0.014	1.783	1.119, 2.840	0.015	1.651	1.032, 2.641	0.037
SII (Optimal cut-off value)									
Low SII	Ref			Ref			Ref		
High SII	2.716	1.748, 4.220	<0.001	2.623	1.685, 4.084	<0.001	2.614	1.676, 4.078	<0.001

Model 1: adjusted for age, mRS score, NIHSS score; Model 2: adjusted for age, mRS score, NIHSS score, hyperlipidemia, previous cerebral infarction, lipid-lowering drugs, and antiplatelet drugs; Model 3: adjusted for age, mRS score, NIHSS score, hyperlipidemia, previous cerebral infarction, lipid-lowering drugs, antiplatelet drugs, SBP, DBP, FBG, uric acid, and eGFR.

SII, Systemic-immune inflammation index; SBP, Systolic blood pressure; DBP, Diastolic blood pressure; FBG, Fasting blood glucose; eGFR, Estimated glomerular filtration rate; mRS, modified Rankin Scale; NIHSS, National Institutes of Health Stroke Scale.

When patients without hyperlipidemia were excluded, the multivariate Cox regression analysis revealed that in the partially adjusted Model 1 and Model 2, higher levels of SII were still significantly associated with the risk of all-cause mortality (all *p* < 0.05). In the fully adjusted Model 3, a one-SD increase in SII was associated with a 20.9% increase in the risk of all-cause mortality (HR = 1.209, 95% CI: 1.051–1.390, *p* = 0.008). Additionally, the risk of all-cause mortality in the high SII group (median-based) was 1.651 times that of the low SII group (HR = 1.651, 95% CI: 1.032–2.641, *p* = 0.037). Notably, the optimal cut-off value-based high SII group showed a more pronounced elevation in mortality risk, with a 2.614-fold higher risk than the low SII group (HR = 2.614, 95% CI: 1.676–4.078, *p* < 0.001) in the fully adjusted model.

### 3.7 Discriminatory ability comparison of SII, PLR and MHR for all-cause mortality

**Figure 2:**
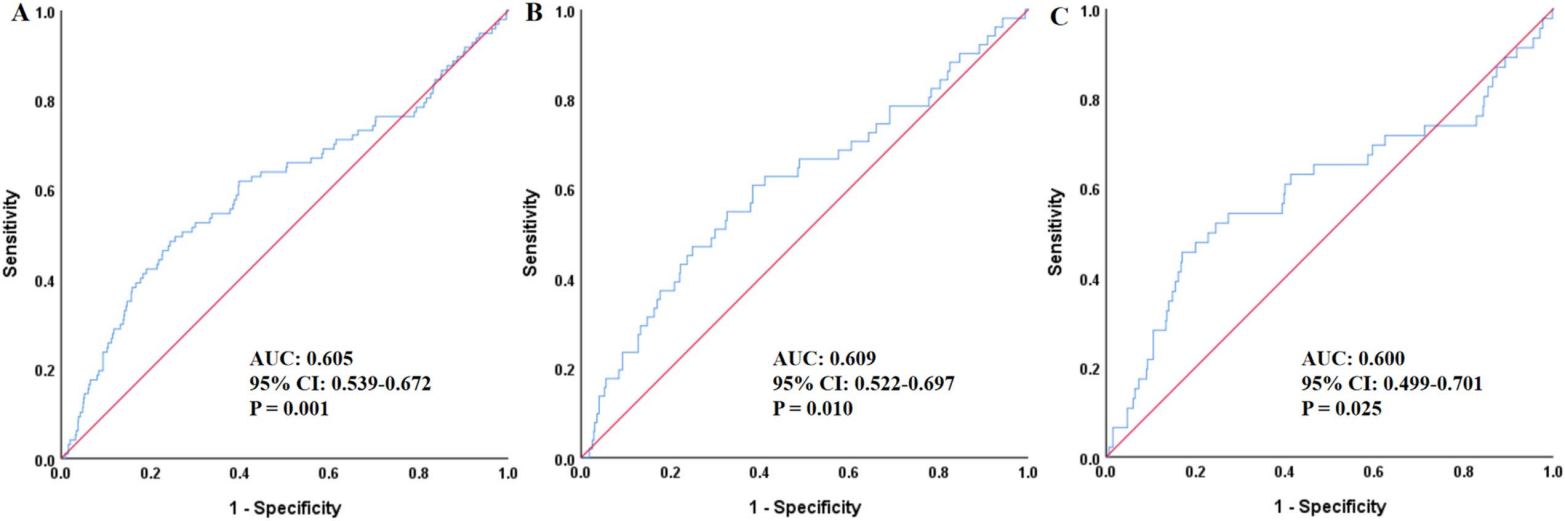
ROC curves assessing the predictive value of SII for all-cause mortality in the total population **(A)**, men **(B)**, and women **(C)**. SII, Systemic-immune inflammation index; ROC, receiver operating characteristic; AUC, area under the curve.

As presented in Figure 2, ROC curve analysis demonstrated that SII held a certain discriminatory ability for the risk of all-cause mortality, not only in the overall population but also in the male and female subgroups. The AUC values were 0.605 (95% CI: 0.539–0.672, *p* = 0.001), 0.609 (95% CI: 0.522–0.697, *p* = 0.010), and 0.600 (95% CI: 0.499–0.701, *p* = 0.025), respectively.

**Figure 3:**
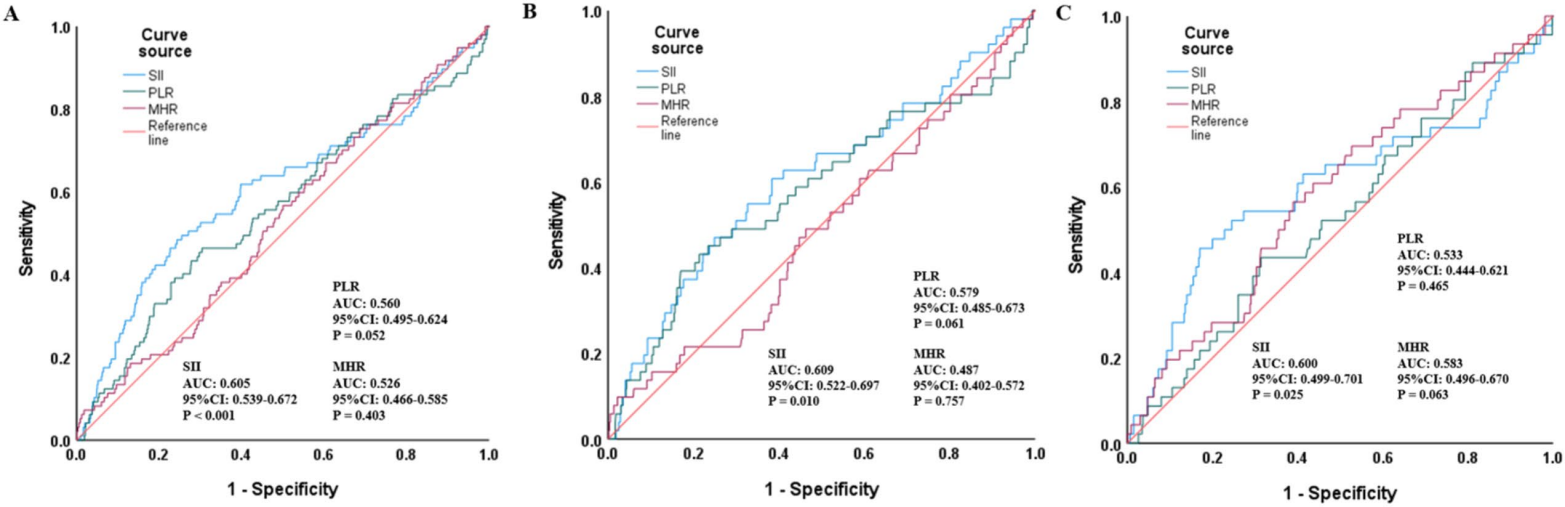
ROC curves assessing the predictive value of SII, PLR, MHR for all-cause mortality in the total population **(A)**, men **(B)**, and women **(C)**. SII, Systemic-immune inflammation index; ROC, receiver operating characteristic; AUC, area under the curve; PLR, platelet-to-lymphocyte ratio; MHR, monocyte to high-density lipoprotein cholesterol ratio.

Descriptive and exploratory ROC curve analysis (Figure 3) was performed to compare the discriminatory ability of SII, PLR and MHR for all-cause mortality in the overall population and gender subgroups. In the overall population, SII had a moderate discriminatory ability with an AUC of 0.605 (95% CI: 0.539–0.672, *p* = 0.001), which was superior to PLR (AUC = 0.560, 95% CI: 0.495–0.624, *p* = 0.052) and MHR (AUC = 0.526, 95% CI: 0.466–0.585, *p* = 0.403). Consistent results were observed in gender subgroups: in male patients, the AUC of SII was 0.609 (95% CI: 0.522–0.697, *p* = 0.010), higher than that of PLR (AUC = 0.579, 95% CI: 0.485–0.673, *p* = 0.061) and MHR (AUC = 0.487, 95% CI: 0.402–0.572, *p* = 0.757); in female patients, the AUC of SII was 0.600 (95% CI: 0.499–0.701, *p* = 0.025), also higher than that of PLR (AUC = 0.533, 95% CI: 0.444–0.621, *p* = 0.465) and MHR (AUC = 0.583, 95% CI: 0.496–0.670, *p* = 0.063). The maximum Youden index of SII was 0.241, corresponding to the optimal SII cut-off value of 986.83, with a sensitivity of 43.3%, a specificity of 78.9%, a positive predictive value of 15.7%, and a negative predictive value of 93.5% at this threshold.

### 3.8 Incremental discriminatory ability of SII combined with NIHSS and mRS scores for all-cause mortality

**Figure 4:**
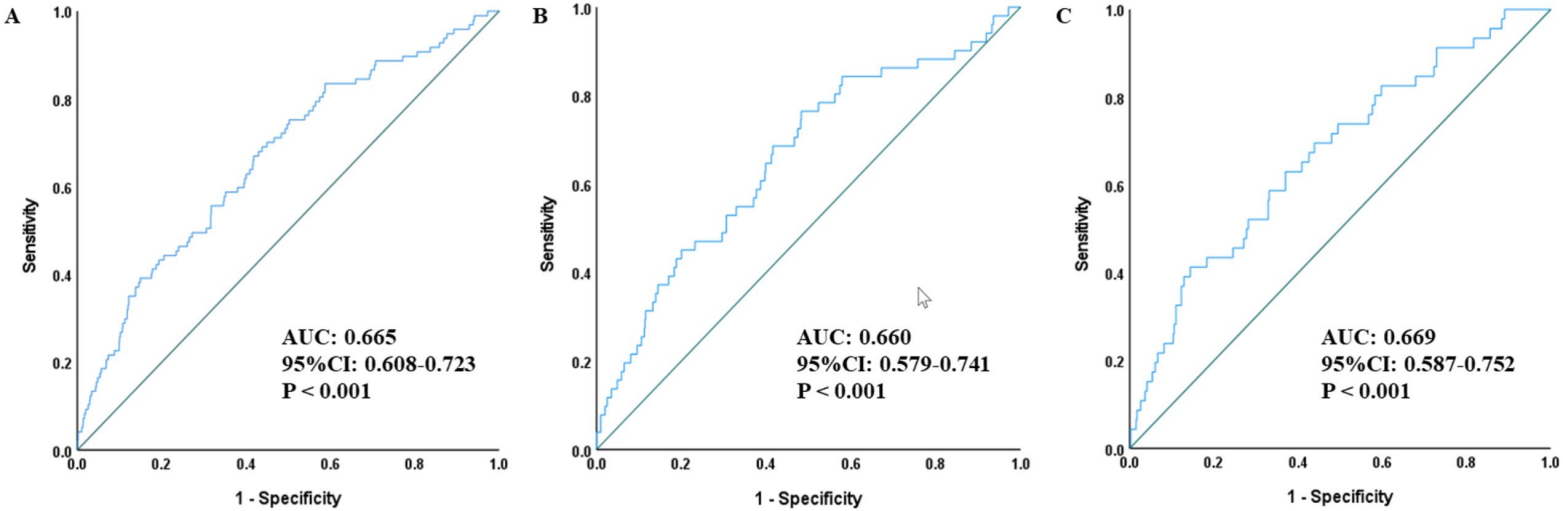
ROC curves for assessing the combined predictive value of SII and NIHSS score for all-cause mortality in the total population **(A)**, men **(B)**, and women **(C)**. SII, Systemic-immune inflammation index; ROC, receiver operating characteristic; AUC, area under the curve; NIHSS, National Institutes of Health Stroke Scale.

**Figure 5:**
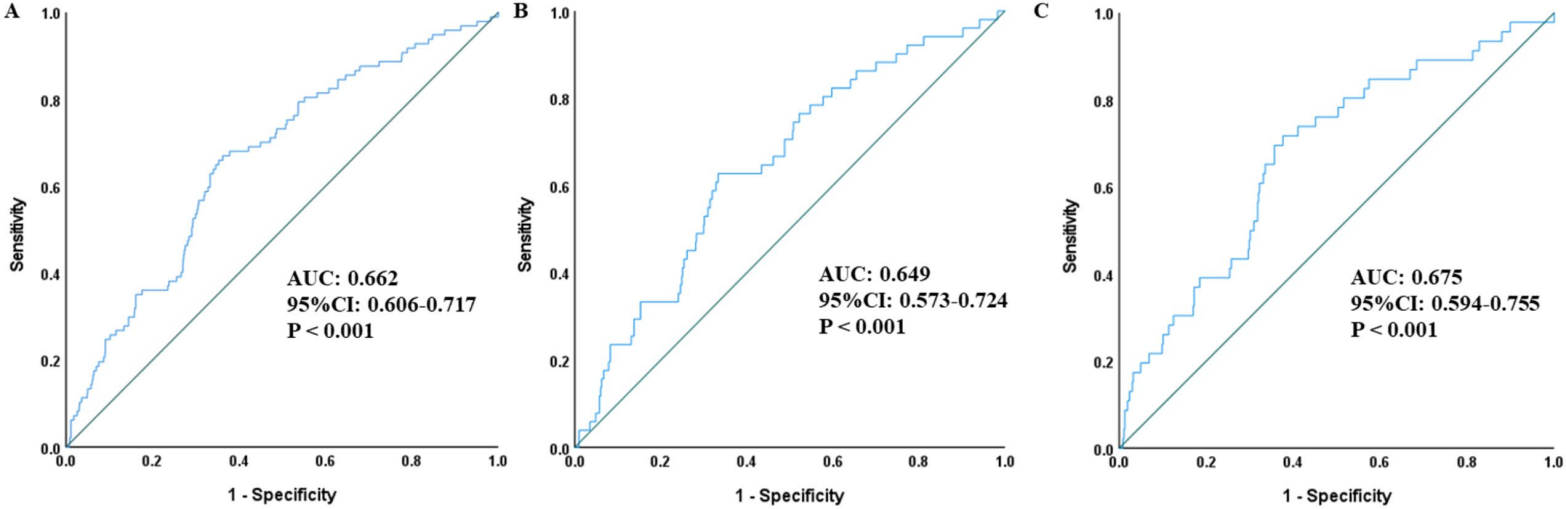
ROC curves for assessing the combined predictive value of SII and mRS score for all-cause mortality in the total population **(A)**, men **(B)**, and women **(C)**. SII, Systemic-immune inflammation index; ROC, receiver operating characteristic; AUC, area under the curve; mRS, modified Rankin Scale.

**Figure 6:**
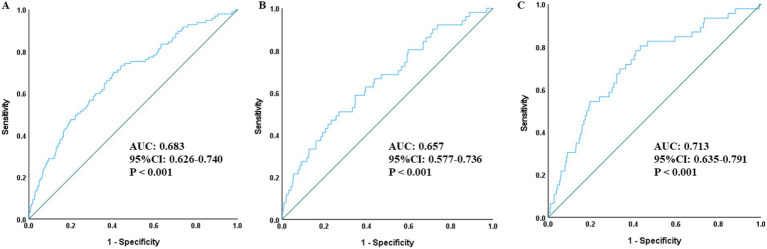
ROC curves for evaluating the combined predictive value of SII with NIHSS score and mRS score for all-cause mortality in the total population **(A)**, men **(B)**, and women **(C)**. SII, Systemic-immune inflammation index; ROC, receiver operating characteristic; AUC, area under the curve; mRS, modified Rankin Scale; NIHSS, National Institutes of Health Stroke Scale.

Descriptive and exploratory ROC curve analysis was further performed to evaluate the incremental discriminatory value of combining SII with NIHSS and mRS scores (Figures 4–6). When SII was combined with NIHSS score (Figure 4), the AUC increased to 0.665 (95% CI: 0.608–0.723, *p* < 0.001) in the overall population, 0.660 in males and 0.669 in females. When SII was combined with mRS score (Figure 5), the AUC reached 0.662 (95% CI: 0.606–0.717, *p* < 0.001) in the overall population, 0.649 in males and 0.675 in females. Notably, the combination of SII with both NIHSS and mRS scores achieved the highest discriminatory ability, with an AUC of 0.683 (95% CI: 0.626–0.740, *p* < 0.001) in the overall population, 0.657 in males and 0.713 in females (Figure 6).

### 3.9 Kaplan–Meier survival curve analysis of SII and all-cause mortality

**Figure 7:**
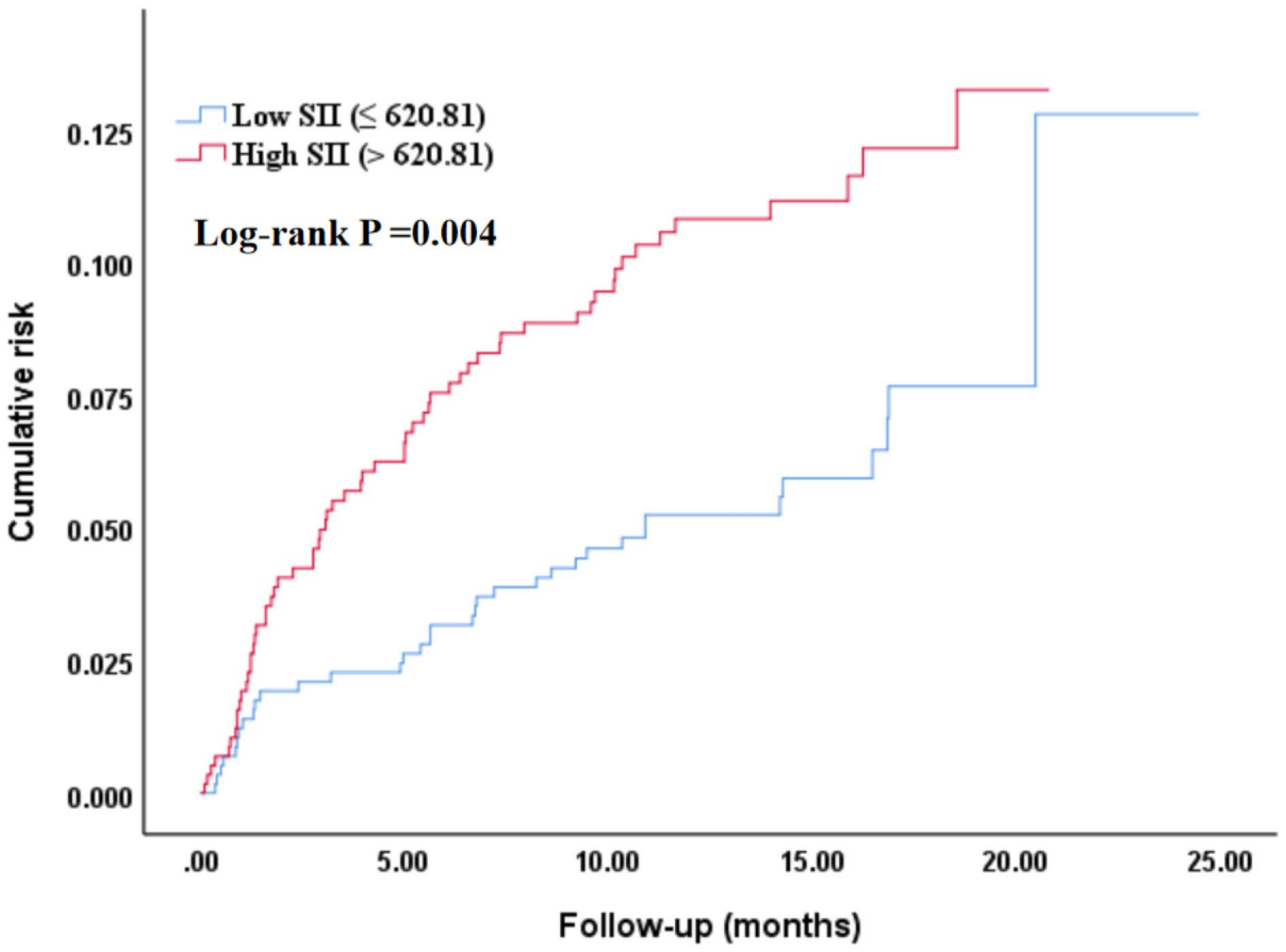
Kaplan–Meier curve assessing the differences in the cumulative risk of all-cause mortality among different SII (median) groups. SII, Systemic-immune inflammation index.

**Figure 8:**
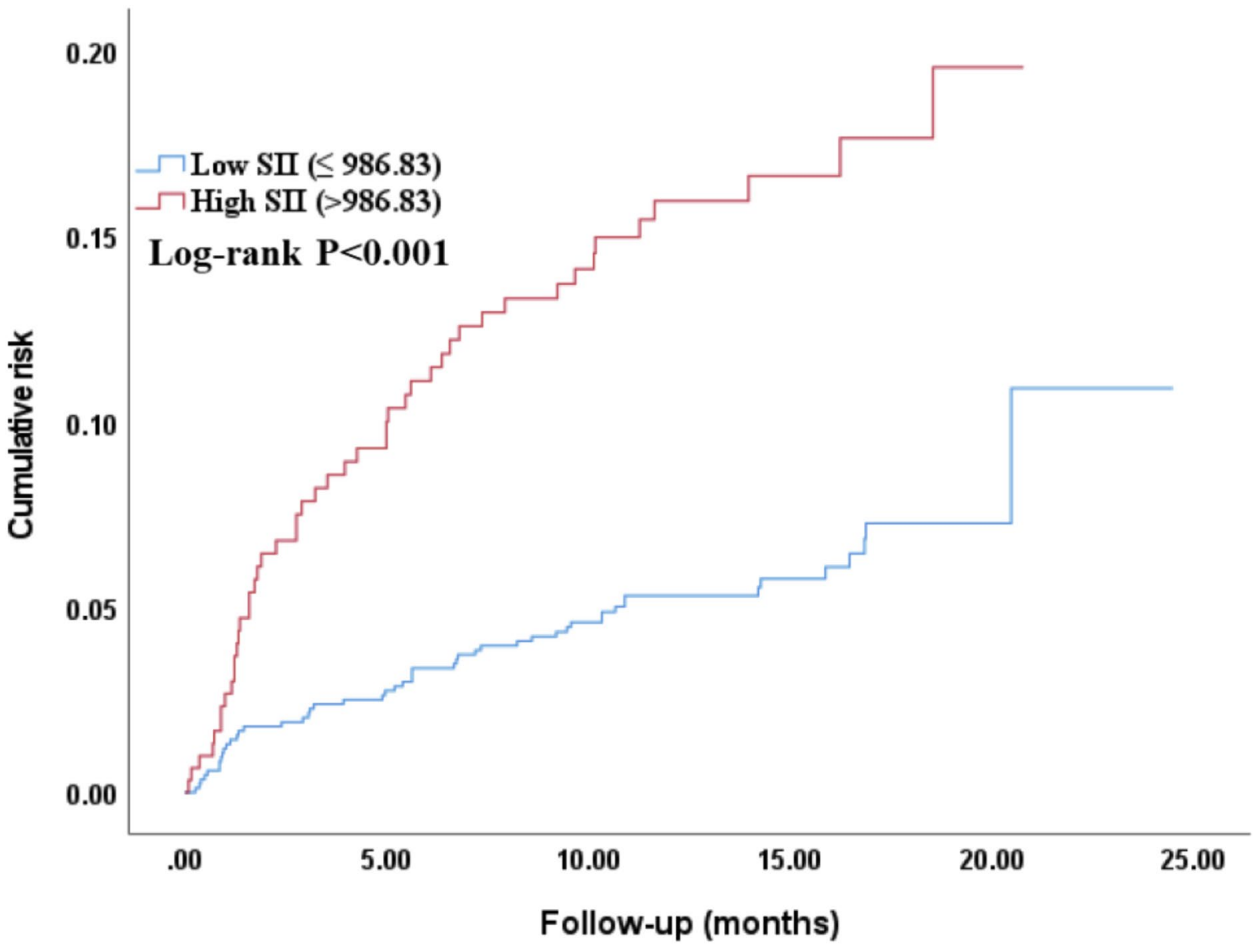
Kaplan–Meier curve assessing the differences in the cumulative risk of all-cause mortality among different SII (optimal cut-off value) groups. SII, Systemic-immune inflammation index.

The Kaplan–Meier survival curve analysis revealed significant differences in the cumulative risk of all-cause mortality among different SII groups. As shown in Figure 7, for the median-based grouping, the cumulative risk of all-cause mortality was significantly higher in the high SII group than in the low SII group (log-rank *p* = 0.004). As shown in Figure 8, for the optimal cut-off value-based grouping, the separation of the survival curves was more pronounced: the high SII group (SII > 986.83) had a markedly higher cumulative mortality risk compared with the low SII group (log-rank *p* < 0.001).

## 4 Discussion

This single-center retrospective cohort study enrolled 1,156 patients with IS. The primary objective of this retrospective cohort study is to analyze the independent association between SII and all-cause mortality in patients with IS, rather than to construct or validate a formal clinical prediction model (27–29). The core findings of this study are detailed as follows: (1) Elevated SII was independently associated with an increased risk of all-cause mortality in IS patients. After adjusting for age, baseline NIHSS score, pre-stroke mRS score, blood pressure, metabolic indicators, and renal function, each 1-SD increment in SII was associated with a 17.2% higher risk of all-cause mortality (HR = 1.172, 95% CI: 1.019–1.348, *p* = 0.026), and patients in the high SII group (SII > 620.81) had a 1.543-fold higher risk of all-cause mortality than those in the low SII group (HR = 1.543, 95% CI: 1.017–2.340, *p* = 0.041); (2) Subgroup analysis revealed that the association between elevated SII and increased all-cause mortality risk was more prominent in patients aged ≥ 70 years, smokers, and those with a history of previous cerebral infarction, and the optimal cut-off value of 986.83 showed wider and more stable prognostic applicability across all subgroups; (3) Sensitivity analysis (excluding patients without hypertension or hyperlipidemia) further verified the stability of the association between SII and all-cause mortality risk, with HR values for per SD increment in SII ranging from 1.170 to 1.209 (*p* < 0.05); (4) Descriptive and exploratory ROC curve analysis showed that SII had a moderate discriminatory ability for all-cause mortality in the overall population (AUC = 0.605), as well as in male and female subgroups, and SII exhibited superior discriminatory ability compared with PLR and MHR. The combination of SII with NIHSS score, mRS score, or both further improved the discriminatory ability for all-cause mortality, with the highest AUC reaching 0.683 when combining all three indicators. Notably, given that the core focus of this study was association analysis rather than predictive model development, the ROC curves and corresponding AUC values were applied solely for descriptive and exploratory analyses to characterize the discriminatory performance of SII with respect to all-cause mortality, and were not used to construct or validate a formal clinical prediction model (30–32). The moderate AUC values further indicate that SII alone is not sufficient for clinical outcome prognostic discrimination, and its clinical value lies in reflecting the systemic inflammatory state and assisting in risk stratification when combined with other clinical indicators. We emphasize that the AUC values herein only reflect the exploratory discriminatory performance of SII in this study cohort, and cannot be directly extrapolated as a predictive tool in independent clinical populations without further external validation. Kaplan–Meier survival curve analysis also confirmed that the cumulative risk of all-cause mortality was significantly higher in the high SII group (log-rank *p* = 0.004 for median-based grouping, log-rank *p* < 0.001 for optimal cut-off value-based grouping). These results suggest that SII, as a composite inflammatory biomarker integrating pro-inflammatory components (neutrophils, platelets) and anti-inflammatory components (lymphocytes), can more comprehensively reflect the imbalance of immune-inflammatory homeostasis than single blood cell indicators and other inflammatory composite indices, and has potential value for clinical risk stratification of IS patients after discharge. It is well recognized that inflammatory status varies significantly across different stages of stroke, and that the level of inflammatory stress undergoes dynamic changes throughout the disease course. All enrolled patients in this study were admitted within 24 h of IS onset and were in the acute or subacute phase during hospitalization. The SII values were calculated based on blood samples collected within 24 h of admission, which effectively reflects the inflammatory and immune status of patients in the early stage of the disease. However, it should be acknowledged that this study did not perform more refined stratification of IS stages, which may have exerted a certain influence on the inflammatory status and potentially confounded the association between SII and all-cause mortality. Given the dynamic evolution of inflammatory responses during stroke progression, the lack of stage-specific stratification may have affected the assessment of inflammatory status and potentially interfered with the correlation between SII and all-cause mortality. For example, the inflammatory response peaks in the acute phase and gradually declines in the convalescent phase, which may lead to variations in the strength of the association between SII and mortality risk across different stages. In future research, we will further optimize the study design and re-evaluate the correlation between SII and all-cause mortality in patients with acute IS by incorporating refined stratification of IS stages (e.g., acute phase, subacute phase, convalescent phase) and dynamically monitoring SII levels at multiple time points. This will help to further clarify the stage-specific correlation between SII and all-cause mortality in patients with acute IS, and improve the accuracy and clinical applicability of SII as a prognostic biomarker.

The unique advantages and innovations of this research are systematically summarized in the following aspects: First, this study focuses on long-term all-cause mortality in patients after discharge, excluding short-term in-hospital mortality, and thus is more aligned with the clinical scenario of real-world follow-up and long-term prognostic management. Second, we adopted a multi-angle and multi-level statistical analysis strategy, including multivariate and multi-model Cox regression analyses adjusted for core clinical prognostic markers (NIHSS and mRS scores), subgroup analyses and sensitivity analyses, to systematically verify the stability and validity of the study results. Third, the study population was recruited from Northeast China, making this a real-world study that provides new evidence to supplement the existing data on populations from different regions. Fourth, this study systematically compared the discriminatory ability of SII with PLR and MHR, and explored the incremental discriminatory value of combining SII with NIHSS and mRS scores, providing more comprehensive evidence for the clinical application of SII in IS risk stratification. Fifth, this study simultaneously verified the prognostic performance of median-based grouping and ROC-derived optimal cut-off value-based grouping, and confirmed that the optimal cut-off value of 986.83 has better prognostic discrimination for all-cause mortality in IS patients, providing a quantifiable threshold for clinical risk stratification.

The underlying mechanisms for the heterogeneity of the association across subgroups are further elaborated as follows. For patients aged ≥ 70 years, immune senescence and impaired inflammatory regulatory capacity may amplify the inflammatory damage mediated by elevated SII (33, 34). For smokers, it can thus be concluded that smoking activates innate and adaptive immune responses, induces M1 macrophage polarization, upregulates proinflammatory cytokines while downregulating anti-inflammatory factors, and simultaneously exacerbates the excessive production of reactive oxygen species (ROS) and reactive nitrogen species (RNS). This leads to the synergistic exacerbation of blood–brain barrier injury and neuronal death by systemic inflammation and oxidative stress, thereby significantly increasing the risk of stroke recurrence and resulting in poorer functional outcomes. The nicotine-induced endothelial dysfunction exerts a synergistic effect with elevated SII, which explains the more prominent association between SII and mortality risk in this population (35–40). For patients with a history of previous cerebral infarction, it can thus be concluded that a history of stroke indicates the presence of chronic neuroinflammation and systemic endothelial dysfunction (e.g., persistent endothelial cell activation, pyroptosis, or endothelial-mesenchymal transition) triggered by atherosclerosis and cerebral ischemia, which forms a vicious cycle of inflammation-vascular injury (41–44). Against this backdrop, SII, as an inflammatory biomarker integrating the dynamic changes in platelets, neutrophils and lymphocytes, can more sensitively reflect the sustained systemic inflammatory burden and immune imbalance status following stroke. The pre-existing vascular lesions lead to poorer tolerance to inflammatory damage in these patients, which explains the strongest association between SII and all-cause mortality in this subgroup (13, 45). For the optimal cut-off value analysis, the present study identified 986.83 as the optimal SII cut-off value for distinguishing all-cause mortality in northern Chinese IS patients via the maximum Youden index, which yielded a specificity of 78.9% and a negative predictive value of 93.5%. Across multivariate Cox regression, subgroup analysis, and sensitivity analysis, stratification by this threshold showed a more robust association with all-cause mortality compared with median-based stratification, with the HR for the high SII group consistently exceeding 2.1. Notably, the statistical significance was retained at the *p* < 0.001 level even after full adjustment for potential confounding factors including NIHSS and mRS scores. The potential clinical utility of this optimal cut-off value is twofold: first, its high specificity and elevated negative predictive value enable clinicians to accurately exclude low-risk patients, thereby avoiding unnecessary medical interventions and optimizing the allocation of healthcare resources; second, it can effectively identify IS patients with an extremely high risk of mortality, who are likely to benefit from intensified anti-inflammatory therapy, stringent control of cardio-cerebrovascular risk factors, and more rigorous follow-up surveillance. In terms of the comparative advantage of SII over other inflammatory biomarkers, our study found that SII had superior discriminatory ability for all-cause mortality in IS patients compared with PLR and MHR. This may be attributed to the fact that SII integrates three key components of the immune-inflammatory response: neutrophils (core pro-inflammatory cells), platelets (mediators of thrombosis and inflammatory amplification), and lymphocytes (core anti-inflammatory and immune regulatory cells). In contrast, PLR only reflects the balance between platelets and lymphocytes, and MHR only reflects the association between monocytes and lipid metabolism, both of which cannot comprehensively characterize the integrated state of systemic immunity and inflammation in IS patients. This finding is consistent with previous studies showing that SII has better prognostic value for adverse outcomes in cardiovascular and cerebrovascular diseases than other single or dual-component inflammatory indices (19, 21). Furthermore, our study confirmed that combining SII with NIHSS and mRS scores can further improve the discriminatory ability for all-cause mortality. This is because SII reflects the systemic inflammatory state, while NIHSS and mRS scores reflect the severity of neurological impairment and residual functional status, respectively. The combination of inflammatory biomarkers and clinical prognostic markers can provide a more comprehensive assessment of the mortality risk in IS patients, which is consistent with the current clinical practice of multi-indicator combined risk stratification. SII is associated with post-stroke mortality via multiple pathways, including systemic inflammatory activation, endothelial dysfunction, and immunometabolic dysregulation. A robust systemic inflammatory response is rapidly elicited in the body following stroke, and an elevated SII reflects enhanced activation of neutrophils and platelets alongside lymphocyte depletion, indicative of immune imbalance and a persistent inflammatory state. Accumulating evidence has demonstrated that indices including SII and the systemic inflammatory response index (SIRI) are involved in the regulation of systemic inflammatory response, and are associated with the development of post-ICH complications, such as SAP (12). The ensuing inflammatory cascade further releases proinflammatory cytokines, which exacerbates tissue damage. A meta-analysis has identified a significant correlation between SII levels and stroke severity (standard mean difference [SMD] = 1.35, 95% CI: 0.48–2.23, *p* = 0.002) (9). Although SII exerts no significant effect on vascular recanalization, the persistent inflammatory state indicated by high SII levels may be associated with impaired survival outcomes by amplifying secondary brain injury and increasing susceptibility to infections. Furthermore, as a reliable surrogate marker for the severity of systemic inflammation, SII has been validated to be independently associated with poor prognosis across a spectrum of diseases (14). After stroke, SII may indirectly increase mortality risk by sustaining a state of chronic low-grade inflammation and compromising tissue repair capacity. Inflammatory activation, as reflected by an elevated SII, is also associated with accelerated vascular endothelial injury. Extant studies have clearly established that inflammatory biomarkers, including SII, participate in the pathological damage of blood vessels, the kidneys, the retina, and peripheral nerves by triggering systemic and local inflammatory cascades. In the context of stroke, endothelial dysfunction can induce blood–brain barrier disruption, aggravated cerebral edema, and microcirculatory impairment, which further exacerbates neurological dysfunction. While the causal link between SII and post-stroke endothelial function has not been directly elucidated in the literature, SII serves as a composite indicator of platelet and neutrophil activation; its elevation signifies the coexistence of prothrombotic and proinflammatory states, which may accelerate the progression of vascular endothelial injury and thereby provide a pathological basis for the observed association between SII and poor prognosis (45). In addition, SII is closely correlated with metabolic abnormalities. Multiple studies have confirmed a significant association between SII levels and metabolic syndrome (MetS) as well as its components (e.g., insulin resistance, obesity) (46, 47), and MetS is a well-recognized major risk factor for stroke. In the acute phase of stroke, pre-existing metabolic disturbances superimposed on stress responses can exacerbate immune cell dysfunction (e.g., lymphopenia, excessive neutrophil activation), thus forming a vicious cycle between inflammation and metabolism. Emerging evidence has suggested that SII and SIRI may act as mediators between cardiometabolic multimorbidity (CMM) and adverse clinical outcomes (48), implicating their bridging role in linking metabolic abnormalities and inflammatory responses. Additionally, in populations with CKM syndrome, SII and SIRI are associated with an increased risk of cardiovascular diseases (15), which suggests that in stroke patients, SII may be associated with compromised recovery potential and long-term survival by amplifying underlying metabolic dysregulation. Consistent with the core findings of this study, accumulating previous evidence has confirmed the significant prognostic value of SII in cerebrovascular diseases, particularly in IS populations. For example, a systematic review and meta-analysis by Huang et al. (9) revealed that elevated SII levels were significantly correlated with increased mortality in stroke patients, which is in line with the core conclusion of our study that higher SII is independently associated with elevated all-cause mortality risk in IS patients. Besides, Jin et al. (49) found in a large cohort of 4,262 stroke patients that elevated SII was associated with an increased risk of all-cause mortality, providing a large-sample foundation for the correlation we verified in the northern Chinese IS population. In a retrospective cohort study involving 40,670 patients, Zhang et al. (50) identified that an SII value higher than 583 was an independent risk factor for adverse IS outcomes, which was highly consistent with the median cut-off value of 620.81 in this study, confirming the stability and clinical universality of SII as a prognostic indicator across different populations.

Current evidence suggests that the following pathophysiological mechanisms may mediate the association between SII and all-cause mortality in IS. Firstly, oxidative stress is closely related to the pathological process of IS. In patients with IS, the imbalance between excessive ROS production and the body’s antioxidant capacity can lead to further deterioration of brain injury (51, 52), SII is not only associated with the severity of stroke but may also affect the risk of death through the oxidative stress pathway (53). Besides, inflammatory factors play a crucial role in the association between the SII and the all-cause mortality risk of patients with IS. By taking into comprehensive consideration multiple inflammatory indicators, such as C-reactive protein (CRP), interleukin-6 (IL-6), ferritin, and oxidative stress markers, we can gain a more thorough understanding of the role of inflammation in the prognosis of stroke. These findings not only contribute to the identification of high-risk patients but also provide a foundation for the development of personalized treatment strategies (9, 54). When exploring the relationship between the SII and all-cause mortality or poor prognosis in patients with IS, the role of inflammatory markers such as CRP cannot be ignored. Multiple studies have shown that elevated CRP levels in AIS are closely related to poor prognosis. For example, a systematic review and meta-analysis demonstrated that elevated CRP levels in AIS patients were significantly associated with poor prognosis at 3 months (55). Additionally, another study indicated that elevated CRP levels were associated with an increased risk of all-cause mortality in patients with AIS (56). In summary, inflammatory markers such as CRP and SII play an important role in the prognosis assessment of patients with AIS. By combining the use of these markers, the prognosis of patients can be predicted more accurately, and guidance can be provided for clinical treatment.

Despite the well-recognized prognostic value of CRP in IS, we did not include CRP in the formal statistical analysis of this study for the following reasons. The data used in this study were derived from a single-center retrospective hospital database, and CRP is not a routine examination item for IS patients in our center. The measurement of CRP was mainly determined by clinicians’ judgment of the patient’s concurrent infection and disease condition, resulting in an extremely high missing rate of 93.7% for CRP data in the overall cohort. Including CRP in the analysis would drastically reduce the sample size available for statistical analysis and introduce significant selection bias, while imputation of the missing CRP data would severely compromise the stability and reliability of the study results. Therefore, we did not include CRP data in the formal analysis of this study. Furthermore, atherosclerosis, as a chronic inflammatory vascular disease, is closely related to various inflammatory markers in its pathological process. In patients with IS, the burden of atherosclerosis is significantly associated with long-term mortality. A previous study have shown that a higher carotid-artery atherosclerosis burden score was associated with a higher all-cause mortality rate in patients (57). This indicates that the severity of atherosclerosis may affect the prognosis of stroke patients by exacerbating the inflammatory response. Atherosclerosis plays a crucial role in patients with IS, especially in the elevation of the all-cause mortality risk. There is evidence suggesting that atherosclerosis is not only one of the major etiologies of IS but also influences the prognosis and mortality risk of patients through indicators such as the SII (19). The association between SII and all-cause mortality has been confirmed, especially in patients with atherosclerotic cardiovascular disease, where an elevated SII is associated with a poorer survival rate (58). As a new-type inflammatory indicator, SII has been demonstrated to be closely associated with the disease severity of patients with large artery atherosclerotic stroke (19). In summary, atherosclerosis may play a mediating role between SII and the prognosis of IS patients by enhancing the inflammatory response. The mechanism underlying the role of cell death in the all-cause mortality risk of patients with IS is a complex process. It involves the interplay of multiple biomarkers and inflammatory indicators. The SII, as an emerging inflammatory biomarker, has been demonstrated to be closely associated with the prognosis of various diseases, including cardiovascular diseases and cancers (59). A previous study have shown that the NLR has a J-shaped relationship with all-cause mortality in stroke patients, indicating that NLR is significant in predicting the long-term prognosis of stroke patients (60). Although these mechanisms can explain the correlation between SII and all-cause mortality in IS, more cellular and animal experiments are still needed for further verification.

Although this study had achieved exciting research findings, it inevitably faced the following limitations: (1) Although the sample size of this study was large, it was a single-center study, and the results might not be applicable to other races, populations, or medical centers; multicenter, large-scale prospective studies are needed to further verify the optimal cut-off value of 986.83 and its prognostic value in different populations. (2) As this was a retrospective study, it was an observational study, and thus genetic association analysis was not included, making it impossible to determine the causal relationship between SII and all-cause mortality; (3) Although this study included a large amount of empirical data and adjusted for multiple confounding factors including baseline NIHSS score and pre-stroke mRS score in the multivariate regression analysis, some important factors were not included due to the nature of the retrospective study, such as genetic association factors, environmental factors, dietary factors, and occupational exposure factors. (4) We acknowledge that inflammatory responses may differ across etiological subtypes of IS. However, due to the retrospective design of this study, the TOAST classification data for more than 50% of the enrolled patients were incomplete, inconsistent, or missing in the medical records. Forced inclusion of TOAST classification as a covariate would have resulted in substantial patient exclusion and potential selection bias. Accordingly, stroke etiological subtypes were not included as an adjusted variable in the analysis, and we were unable to adjust for TOAST classification in the current regression models. In future research, we will conduct prospective studies with standardized and clearly defined TOAST classification for all enrolled patients, to further explore the impact of different TOAST subtypes on the association between SII and all-cause mortality in patients with IS. (5) We fully acknowledge that acute-phase therapeutic interventions for IS, such as surgery and vascular recanalization, can provoke a profound inflammatory and stress response, which may substantially confound the association between baseline SII and clinical outcomes. In this study, the SII index we calculated was mainly based on the first hematological test results obtained after patient admission, which were mostly performed before or at an early stage of any intervention, thereby minimizing the impact of these treatments on SII. However, due to the limitations inherent in retrospective studies, we did not systematically collect and analyze the specific acute-phase treatment modalities received by patients, which may lead to residual confounding bias in the association between SII and all-cause mortality in IS patients. In future studies, we will further refine the study design by comprehensively collecting specific acute-phase interventions and dynamically monitoring SII levels at multiple time points to explore the true relationship between SII and all-cause mortality. (6) We did not include CRP, a classic inflammatory biomarker, in the formal analysis of this study due to the extremely high missing rate (93.7%) in the retrospective database, which may lead to insufficient comprehensive evaluation of the inflammatory state of IS patients. The lack of systematic collection and analysis of CRP data also limits the further exploration of the combined prognostic value of SII and classic inflammatory markers in this study. In future prospective studies, we will systematically collect inflammatory markers including CRP, and perform a combined analysis with SII to further improve the prognostic value of SII for all-cause mortality in IS patients. (7) The ROC curve analysis showed that the AUC of SII alone was 0.605, indicating that SII alone has limited discriminatory ability for all-cause mortality and cannot be used as an independent prognostic tool. Its clinical value lies in assisting risk stratification when combined with other clinical indicators such as NIHSS and mRS scores. Besides, in strict accordance with the core positioning of this study focusing on association analysis rather than predictive model development, all ROC curve and AUC results were only used for descriptive and exploratory analysis to characterize the discriminatory ability of biomarkers for the study endpoint, rather than for establishing or validating a formal clinical predictive model. The moderate AUC values further indicate that SII alone is not sufficient for clinical outcome prognostic discrimination, and its clinical value lies in reflecting the systemic inflammatory state and assisting in risk stratification when combined with other clinical indicators. (8) The main outcome of this study was only all-cause mortality, and other cerebrovascular events such as cerebral infarction recurrence and cerebral hemorrhage were not further investigated, so the results might not fully reflect the impact of SII on the poor prognosis of cerebral infarction patients. In addition, this study only measured the SII level at admission, and did not dynamically monitor the changes of SII during hospitalization and follow-up, which may have a higher prognostic value for the long-term outcome of IS patients.

## 5 Conclusion

This study confirmed that higher levels of SII are significantly associated with a higher risk of all-cause mortality in IS patients, and this association remained stable after adjusting for core clinical prognostic markers including baseline NIHSS score and pre-stroke mRS score. SII showed better discriminatory ability for all-cause mortality in IS patients than other inflammatory composite indices (PLR and MHR), and the combination of SII with NIHSS and mRS scores provided incremental discriminatory value for all-cause mortality risk assessment. This suggests that in the daily diagnosis and treatment of IS patients, attention should be paid to SII and the involvement of inflammatory and immune factors, and the optimal cut-off value of 986.83 can be used to identify high-risk patients for early targeted intervention. In the future, research designs should be further improved by combining artificial intelligence, bioinformatics, computational medicine, and cell and animal experiments to make up for the deficiencies of this study. We will also conduct prospective multicenter studies to systematically collect classic inflammatory markers including CRP, perform standardized TOAST classification for all enrolled patients, and dynamically monitor SII levels at multiple time points, to further verify the clinical applicability of the optimal cut-off value and improve the prognostic value of SII for all-cause mortality in IS patients, so as to provide better clinical promotion for reducing the high mortality rate and premature death of IS patients.

## Data Availability

The raw data supporting the conclusions of this article will be made available by the authors, without undue reservation.

## References

[1] Zhu W He X Huang D Jiang Y Hong W Ke S . Global and regional burden of ischemic stroke disease from 1990 to 2021: an age-period-cohort analysis. Transl Stroke Res. (2025) 16:1474–85. doi: 10.1007/s12975-024-01319-9, 39699770

[2] Yu MY Caprio FZ Bernstein RA. Cardioembolic stroke. Neurol Clin. (2024) 42:651–61. doi: 10.1016/j.ncl.2024.03.002, 38937034

[3] Satue E Vila-Corcoles A Ochoa-Gondar O de Diego C Forcadell MJ Rodriguez-Blanco T . Incidence and risk conditions of ischemic stroke in older adults. Acta Neurol Scand. (2016) 134:250–7. doi: 10.1111/ane.12535, 26592375

[4] Jibril KA Kuiper KJ Nawaz B Naess H Fromm A Øygarden H . Burden of coronary artery disease as a predictor of new vascular events and mortality in patients with ischemic stroke: insights from the Norwegian stroke in the young study. J Am Heart Assoc. (2025) 14:e038899. doi: 10.1161/JAHA.124.038899, 40079310 PMC12132760

[5] Boehme AK Esenwa C Elkind MS. Stroke risk factors, genetics, and prevention. Circ Res. (2017) 120:472–95. doi: 10.1161/CIRCRESAHA.116.308398, 28154098 PMC5321635

[6] Jaffre A Ruidavets JB Calviere L Viguier A Ferrieres J Larrue V. Risk factor profile by etiological subtype of ischemic stroke in the young. Clin Neurol Neurosurg. (2014) 120:78–83. doi: 10.1016/j.clineuro.2014.02.017, 24731581

[7] Nakamura A Otani K Shichita T. Lipid mediators and sterile inflammation in ischemic stroke. Int Immunol. (2020) 32:719–25. doi: 10.1093/intimm/dxaa027, 32300780

[8] Yang YL Wu CH Hsu PF Chen SC Huang SS Chan WL . Systemic immune-inflammation index (SII) predicted clinical outcome in patients with coronary artery disease. Eur J Clin Investig. (2020) 50:e13230. doi: 10.1111/eci.13230, 32291748

[9] Huang YW Yin XS Li ZP. Association of the systemic immune-inflammation index (SII) and clinical outcomes in patients with stroke: a systematic review and meta-analysis. Front Immunol. (2022) 13:1090305. doi: 10.3389/fimmu.2022.1090305, 36591305 PMC9797819

[10] Ma F Li L Xu L Wu J Zhang A Liao J . The relationship between systemic inflammation index, systemic immune-inflammatory index, and inflammatory prognostic index and 90-day outcomes in acute ischemic stroke patients treated with intravenous thrombolysis. J Neuroinflammation. (2023) 20:220. doi: 10.1186/s12974-023-02890-y, 37777768 PMC10543872

[11] Stephenson SS Kravchenko G Gawron-Skarbek A Kostka T Sołtysik BK. Association between immuno-nutritional biomarkers and mortality in hospitalized geriatric population. Front Immunol. (2025) 16:1692551. doi: 10.3389/fimmu.2025.1692551, 41357173 PMC12676944

[12] Wang RH Wen WX Jiang ZP du ZP Ma ZH Lu AL . The clinical value of neutrophil-to-lymphocyte ratio (NLR), systemic immune-inflammation index (SII), platelet-to-lymphocyte ratio (PLR) and systemic inflammation response index (SIRI) for predicting the occurrence and severity of pneumonia in patients with intracerebral hemorrhage. Front Immunol. (2023) 14:1115031. doi: 10.3389/fimmu.2023.1115031, 36860868 PMC9969881

[13] Cheng W Bu X Xu C Wen G Kong F Pan H . Higher systemic immune-inflammation index and systemic inflammation response index levels are associated with stroke prevalence in the asthmatic population: a cross-sectional analysis of the NHANES 1999-2018. Front Immunol. (2023) 14:1191130. doi: 10.3389/fimmu.2023.1191130, 37600830 PMC10436559

[14] Luo J Qin X Zhang X Zhang Y Yuan F Shi W . Prognostic implications of systemic immune-inflammation index in myocardial infarction patients with and without diabetes: insights from the NOAFCAMI-SH registry. Cardiovasc Diabetol. (2024) 23:41. doi: 10.1186/s12933-024-02129-x, 38254086 PMC10804591

[15] Pengfei C Lui M Zhang L Chen C Wang T Ailin H . Association of SII and SIRI with incidence of cardiovascular disease in cardiovascular-kidney-metabolic syndrome: a prospective cohort study. Front Nutr. (2025) 12:1661826. doi: 10.3389/fnut.2025.1661826, 41368184 PMC12683910

[16] Cao Y Li P Zhang Y Qiu M Li J Ma S . Association of systemic immune inflammatory index with all-cause and cause-specific mortality in hypertensive individuals: results from NHANES. Front Immunol. (2023) 14:1087345. doi: 10.3389/fimmu.2023.1087345, 36817427 PMC9932782

[17] Zhang Z Zhang D Cai X Yang Y Sun J Niu G . Association of Systemic Inflammatory Markers with Cerebral Small Vessel Disease Progression: a community-based prospective study. Neurology. (2026) 106:e214711. doi: 10.1212/WNL.0000000000214711, 41687047

[18] Jiang L Cai X Yao D Jing J Mei L Yang Y . Association of inflammatory markers with cerebral small vessel disease in community-based population. J Neuroinflammation. (2022) 19:106. doi: 10.1186/s12974-022-02468-0, 35513834 PMC9072153

[19] Liu K Yang L Liu Y Zhang Y Zhu J Zhang H . Systemic immune-inflammation index (SII) and neutrophil-to-lymphocyte ratio (NLR): a strong predictor of disease severity in large-artery atherosclerosis (LAA) stroke patients. J Inflamm Res. (2025) 18:195–202. doi: 10.2147/JIR.S500474, 39802522 PMC11724665

[20] Cui Z Kuang S Yang X Wang Y Gu S Li H . Predictive value of the systemic immune inflammation (SII) index for stroke-associated pneumonia. Brain Behav. (2023) 13:e3302. doi: 10.1002/brb3.3302, 37938870 PMC10726822

[21] Chen Y Xie K Han Y Xu Q Zhao X. An easy-to-use nomogram based on SII and SIRI to predict in-hospital mortality risk in elderly patients with acute myocardial infarction. J Inflamm Res. (2023) 16:4061–71. doi: 10.2147/JIR.S427149, 37724318 PMC10505402

[22] Zhang Y Liu W Yu H Chen Z Zhang C Ti Y . Value of the systemic immune-inflammatory index (SII) in predicting the prognosis of patients with Peripartum cardiomyopathy. Front Cardiovasc Med. (2022) 9:811079. doi: 10.3389/fcvm.2022.811079, 35252391 PMC8891526

[23] Mancia G Kreutz R Brunström M Burnier M Grassi G Januszewicz A . 2023 ESH guidelines for the management of arterial hypertension the task force for the management of arterial hypertension of the European Society of Hypertension: endorsed by the International Society of Hypertension (ISH) and the European renal association (ERA). J Hypertens. (2023) 41:1874–2071. doi: 10.1097/HJH.0000000000003480 37345492

[24] American Diabetes Association Professional Practice Committee. 2. Diagnosis and classification of diabetes: standards of care in diabetes-2025. Diabetes Care. (2025) 48:S27–49. doi: 10.2337/dc25-S002 39651986 PMC11635041

[25] Li JJ Zhao SP Zhao D Lu GP Peng DQ Liu J . 2023 Chinese guideline for lipid management. Front Pharmacol. (2023) 14:1190934. doi: 10.3389/fphar.2023.1190934, 37711173 PMC10498001

[26] Hu B Yang XR Xu Y Sun YF Sun C Guo W . Systemic immune-inflammation index predicts prognosis of patients after curative resection for hepatocellular carcinoma. Clin Cancer Res. (2014) 20:6212–22. doi: 10.1158/1078-0432.CCR-14-0442, 25271081

[27] Collins GS Dhiman P Ma J Schlussel MM Archer L van Calster B . Evaluation of clinical prediction models (part 1): from development to external validation. BMJ. (2024) 384:e074819. doi: 10.1136/bmj-2023-074819, 38191193 PMC10772854

[28] Riley RD Archer L Snell KIE Ensor J Dhiman P Martin GP . Evaluation of clinical prediction models (part 2): how to undertake an external validation study. BMJ. (2024) 384:e074820. doi: 10.1136/bmj-2023-074820, 38224968 PMC10788734

[29] Riley RD Snell KIE Archer L Ensor J Debray TPA van Calster B . Evaluation of clinical prediction models (part 3): calculating the sample size required for an external validation study. BMJ. (2024) 384:e074821. doi: 10.1136/bmj-2023-074821, 38253388 PMC11778934

[30] Zou KH O'Malley AJ Mauri L. Receiver-operating characteristic analysis for evaluating diagnostic tests and predictive models. Circulation. (2007) 115:654–7. doi: 10.1161/CIRCULATIONAHA.105.594929 17283280

[31] Cook NR. Use and misuse of the receiver operating characteristic curve in risk prediction. Circulation. (2007) 115:928–35. doi: 10.1161/CIRCULATIONAHA.106.672402 17309939

[32] Verbakel JY Steyerberg EW Uno H de Cock B Wynants L Collins GS . ROC curves for clinical prediction models part 1. ROC plots showed no added value above the AUC when evaluating the performance of clinical prediction models. J Clin Epidemiol. (2020) 126:207–16. doi: 10.1016/j.jclinepi.2020.01.028, 32712176

[33] Hearps AC Martin GE Angelovich TA Cheng WJ Maisa A Landay AL . Aging is associated with chronic innate immune activation and dysregulation of monocyte phenotype and function. Aging Cell. (2012) 11:867–75. doi: 10.1111/j.1474-9726.2012.00851.x, 22708967

[34] Lewis ED Wu D Meydani SN. Age-associated alterations in immune function and inflammation. Prog Neuro-Psychopharmacol Biol Psychiatry. (2022) 118:110576. doi: 10.1016/j.pnpbp.2022.110576, 35588939

[35] Zhang Z Wang M Gill D Liu X. Genetically predicted smoking and drinking and functional outcome after ischemic stroke. Neurology. (2022) 99:e2693–8. doi: 10.1212/WNL.0000000000201291, 36130842

[36] Saaoud F Shao Y Cornwell W Wang H Rogers TJ Yang X. Cigarette smoke modulates inflammation and immunity via reactive oxygen species-regulated trained immunity and trained tolerance mechanisms. Antioxid Redox Signal. (2023) 38:1041–69. doi: 10.1089/ars.2022.0087, 36017612 PMC10171958

[37] Saint-André V Charbit B Biton A Rouilly V Possémé C Bertrand A . Smoking changes adaptive immunity with persistent effects. Nature. (2024) 626:827–35. doi: 10.1038/s41586-023-06968-8, 38355791 PMC10881394

[38] Amerio E Sparano F Muñoz-Sanz A Valles C Nart J Monje A. Effects of smoking on macrophage polarization in peri-implantitis lesions. Clin Oral Implants Res. (2025) 36:1017–29. doi: 10.1111/clr.14448, 40371910 PMC12319378

[39] Gerdanovics A Stănescu IC Mîrza CM Dogaru GB Nicula CA Boarescu P-M . Proinflammatory risk factors in patients with ischemic stroke: a systematic review and meta-analysis. Antioxidants. (2025) 14:1229. doi: 10.3390/antiox14101229, 41154538 PMC12561542

[40] Sadanandan N Cozene B Park YJ Farooq J Kingsbury C Wang ZJ . Pituitary adenylate cyclase-activating polypeptide: a potent therapeutic agent in oxidative stress. Antioxidants (Basel). (2021) 10:354. doi: 10.3390/antiox10030354, 33653014 PMC7996859

[41] Endres M Moro MA Nolte CH Dames C Buckwalter MS Meisel A. Immune pathways in etiology, acute phase, and chronic sequelae of ischemic stroke. Circ Res. (2022) 130:1167–86. doi: 10.1161/CIRCRESAHA.121.319994, 35420915

[42] Var SR Shetty AV Grande AW Low WC Cheeran MC. Microglia and macrophages in neuroprotection, neurogenesis, and emerging therapies for stroke. Cells. (2021) 10:3555. doi: 10.3390/cells10123555, 34944064 PMC8700390

[43] Mihaela A Cercel A Doeppner TR Hermann DM Surugiu R Pirscoveanu DFV . Review: systemic inflammation after stroke. Therapy and perspective. Geroscience. 48:2179–200. doi: 10.1007/s11357-025-02070-1, 41501546 PMC12972390

[44] Liu M Wang D Qi C Zou M Song J Li L . Brain ischemia causes systemic Notch1 activity in endothelial cells to drive atherosclerosis. Immunity. (2024) 57:2157–2172.e7. doi: 10.1016/j.immuni.2024.07.002, 39079536

[45] Rao PP Mishra S Gupta J Vyas M Babu MR. Inflammation and immune biomarkers: new frontiers in understanding and managing diabetes complications. Inflammopharmacology. (2025) 33:6507–34. doi: 10.1007/s10787-025-01996-4, 41045345

[46] Zhao Y Shao W Zhu Q Zhang R Sun T Wang B . Association between systemic immune-inflammation index and metabolic syndrome and its components: results from the National Health and nutrition examination survey 2011-2016. J Transl Med. (2023) 21:691. doi: 10.1186/s12967-023-04491-y, 37794370 PMC10548719

[47] Ramezankhani A Tohidi M Hadaegh F. Association between the systemic immune-inflammation index and metabolic syndrome and its components: results from the multi-ethnic study of atherosclerosis (MESA). Cardiovasc Diabetol. (2025) 24:78. doi: 10.1186/s12933-025-02629-4, 39955525 PMC11830208

[48] Hu S Song J Jiang H Wei B Wang H. Association between the dietary index for gut microbiota and cardiometabolic multimorbidity: systemic immune-inflammation index and systemic inflammatory response index. Front Nutr. (2025) 12:1591799. doi: 10.3389/fnut.2025.1591799, 40538590 PMC12176575

[49] Jin Z Wu Q Chen S Gao J Li X Zhang X . The associations of two novel inflammation indexes, SII and SIRI with the risks for cardiovascular diseases and all-cause mortality: a ten-year follow-up study in 85,154 individuals. J Inflamm Res. (2021) 14:131–40. doi: 10.2147/JIR.S283835, 33500649 PMC7822090

[50] Zhang F Niu M Wang L Liu Y Shi L Cao J . Corrigendum: systemic-immune-inflammation index as a promising biomarker for predicting perioperative ischemic stroke in older patients who underwent non-cardiac surgery. Front Aging Neurosci. (2022) 14:1101574. doi: 10.3389/fnagi.2022.1101574, 36570527 PMC9773973

[51] Kahles T Brandes RP. NADPH oxidases as therapeutic targets in ischemic stroke. Cell Mol Life Sci. (2012) 69:2345–63. doi: 10.1007/s00018-012-1011-8, 22618244 PMC11114534

[52] Chen H Yoshioka H Kim GS Jung JE Okami N Sakata H . Oxidative stress in ischemic brain damage: mechanisms of cell death and potential molecular targets for neuroprotection. Antioxid Redox Signal. (2011) 14:1505–17. doi: 10.1089/ars.2010.3576, 20812869 PMC3061196

[53] Hou D Wang C Luo Y Ye X Han X Feng Y . Systemic immune-inflammation index (SII) but not platelet-albumin-bilirubin (PALBI) grade is associated with severity of acute ischemic stroke (AIS). Int J Neurosci. (2021) 131:1203–8. doi: 10.1080/00207454.2020.1784166, 32546038

[54] Huang S Xie W Gao Y Jin Y Chen Y Zhou G . A role for systemic inflammation in stroke-associated infection and the long-term prognosis of acute ischemic stroke: a mediation analysis. J Inflamm Res. (2024) 17:6533–45. doi: 10.2147/JIR.S474344, 39318992 PMC11420892

[55] Jiang J Tan C Zhou W Peng W Zhou X du J . Plasma C-reactive protein level and outcome of acute ischemic stroke patients treated by intravenous thrombolysis: a systematic review and Meta-analysis. Eur Neurol. (2021) 84:145–50. doi: 10.1159/000514099, 33839726

[56] Yu B Yang P Xu X Shao L. C-reactive protein for predicting all-cause mortality in patients with acute ischemic stroke: a meta-analysis. Biosci Rep. (2019) 39:BSR20181135. doi: 10.1042/BSR20181135, 30718369 PMC6379508

[57] Lainelehto K Pienimäki JP Savilahti S Huhtala H Numminen H Putaala J. Cervicocerebral atherosclerosis burden increases long-term mortality in patients with ischemic stroke or transient ischemic attack. J Am Heart Assoc. (2024) 13:e032938. doi: 10.1161/JAHA.123.032938, 38842273 PMC11255707

[58] He L Xie X Xue J Xie H Zhang Y. Association of the systemic immune-inflammation index with all-cause mortality in patients with arteriosclerotic cardiovascular disease. Front Cardiovasc Med. (2022) 9:952953. doi: 10.3389/fcvm.2022.952953, 36172591 PMC9510918

[59] Xia Y Xia C Wu L Li Z Li H Zhang J. Systemic immune inflammation index (SII), system inflammation response index (SIRI) and risk of all-cause mortality and cardiovascular mortality: a 20-year follow-up cohort study of 42,875 US adults. J Clin Med. (2023) 12:1128. doi: 10.3390/jcm12031128, 36769776 PMC9918056

[60] Chen Y Lv T Lin W Meng T Sui Y Chen S. J-shaped association of neutrophil-to-lymphocyte ratio with all-cause mortality and linear association with cardiovascular mortality in stroke survivors. Front Neurol. (2025) 16:1473802. doi: 10.3389/fneur.2025.1473802, 40098686 PMC11911178

